# Effect of Transcranial Low-Level Light Therapy vs Sham Therapy Among Patients With Moderate Traumatic Brain Injury: A Randomized Clinical Trial

**DOI:** 10.1001/jamanetworkopen.2020.17337

**Published:** 2020-09-14

**Authors:** Maria Gabriela Figueiro Longo, Can Ozan Tan, Suk-tak Chan, Jonathan Welt, Arman Avesta, Eva Ratai, Nathaniel David Mercaldo, Anastasia Yendiki, Jacqueline Namati, Isabel Chico-Calero, Blair A. Parry, Lynn Drake, Rox Anderson, Terry Rauch, Ramon Diaz-Arrastia, Michael Lev, Jarone Lee, Michael Hamblin, Benjamin Vakoc, Rajiv Gupta

**Affiliations:** 1 Department of Radiology, Massachusetts General Hospital, Harvard Medical School, Boston; 2 Spaulding Rehabilitation Hospital, Boston, Massachusetts; 3 Athinoula A. Martinos Center for Biomedical Imaging, Boston, Massachusetts; 4 School of Medicine, University of Michigan, Ann Arbor; 5 Department of Radiology, Yale School of Medicine, New Haven, Connecticut; 6 Office of Secretary of Defense, Department of Defense, Washington, DC; 7 Department of Neurology, University of Pennsylvania, Philadelphia

## Abstract

**Importance:**

Preclinical studies have shown that transcranial near-infrared low-level light therapy (LLLT) administered after traumatic brain injury (TBI) confers a neuroprotective response.

**Objectives:**

To assess the feasibility and safety of LLLT administered acutely after a moderate TBI and the neuroreactivity to LLLT through quantitative magnetic resonance imaging metrics and neurocognitive assessment.

**Design, Setting, and Participants:**

A randomized, single-center, prospective, double-blind, placebo-controlled parallel-group trial was conducted from November 27, 2015, through July 11, 2019. Participants included 68 men and women with acute, nonpenetrating, moderate TBI who were randomized to LLLT or sham treatment. Analysis of the response-evaluable population was conducted.

**Interventions:**

Transcranial LLLT was administered using a custom-built helmet starting within 72 hours after the trauma. Magnetic resonance imaging was performed in the acute (within 72 hours), early subacute (2-3 weeks), and late subacute (approximately 3 months) stages of recovery. Clinical assessments were performed concomitantly and at 6 months via the Rivermead Post-Concussion Questionnaire (RPQ), a 16-item questionnaire with each item assessed on a 5-point scale ranging from 0 (no problem) to 4 (severe problem).

**Main Outcomes and Measures:**

The number of participants to successfully and safely complete LLLT without any adverse events within the first 7 days after the therapy was the primary outcome measure. Secondary outcomes were the differential effect of LLLT on MR brain diffusion parameters and RPQ scores compared with the sham group.

**Results:**

Of the 68 patients who were randomized (33 to LLLT and 35 to sham therapy), 28 completed at least 1 LLLT session. No adverse events referable to LLLT were reported. Forty-three patients (22 men [51.2%]; mean [SD] age, 50.49 [17.44] years]) completed the study with at least 1 magnetic resonance imaging scan: 19 individuals in the LLLT group and 24 in the sham treatment group. Radial diffusivity (RD), mean diffusivity (MD), and fractional anisotropy (FA) showed significant time and treatment interaction at 3-month time point (RD: 0.013; 95% CI, 0.006 to 0.019; *P* < .001; MD: 0.008; 95% CI, 0.001 to 0.015; *P* = .03; FA: −0.018; 95% CI, −0.026 to −0.010; *P* < .001).The LLLT group had lower RPQ scores, but this effect did not reach statistical significance (time effect *P* = .39, treatment effect *P* = .61, and time × treatment effect *P* = .91).

**Conclusions and Relevance:**

In this randomized clinical trial, LLLT was feasible in all patients and did not exhibit any adverse events. Light therapy altered multiple diffusion tensor parameters in a statistically significant manner in the late subacute stage. This study provides the first human evidence to date that light therapy engages neural substrates that play a role in the pathophysiologic factors of moderate TBI and also suggests diffusion imaging as the biomarker of therapeutic response.

**Trial Registration:**

ClinicalTrials.gov Identifier: NCT02233413

## Key Points

**Question:**

Is near-infrared low-level light therapy (LLLT) feasible and safe after moderate traumatic brain injury, and does LLLT affect the brain and exhibit neuroreactivity?

**Findings:**

In this randomized clinical trial including 68 patients with moderate traumatic brain injury who were randomized to receive LLLT or sham therapy, 28 patients completed at least 1 LLLT session without any reported adverse events. In the late subacute stage, there were statistically significant differences in the magnetic resonance imaging–derived diffusion parameters of the white matter tracts between the sham- and light-treated groups, demonstrating neuroreactivity of LLLT.

**Meaning:**

The results of this clinical trial show that transcranial LLLT is feasible, safe, and affects the brain in a measurable manner.

## Teaser

This randomized clinical trial evaluates the effect of near-infrared low-level light therapy in patients with traumatic brain injury from the time of injury until 3 months after the injury.

## Introduction

Traumatic brain injury (TBI), a major cause of death and disability, is a significant public health problem in the US and worldwide.^1,2,3^ Traumatic brain injury is defined as an external force-induced injury that may impair normal brain functions, such as memory, movement, sensation, and emotions. Such impairments, which are generally underrated and underreported, may have highly variable clinical presentation.^4^ Traumatic axonal injury is one of the primary pathophysiologic consequences of impact and acceleration injuries to the brain.^5^ White matter tracts are particularly vulnerable to such injury, which may manifest as chemical and mechanical changes in the affected neurons. While these changes could trigger cell apoptosis and consequent neural disconnection, they may also resolve, leading to partial remyelination or even full recovery.^6,7^ Thus, therapies that can induce recovery of myelin in axons after head trauma have been a target of research for several years.^8^

Low-level light therapy (LLLT) uses near-infrared (NIR) light, typically in the 600- to 1100-nm wavelength range, and is believed to elicit biostimulation mediated by mitochondrial light absorption. Specifically, cytochrome C oxidase, a large trans-membrane protein complex that is a part of the respiratory electron transport chain, is thought to absorb the light and upregulate adenosine triphosphate production.^9^ The NIR may also upregulate messenger molecules, including reactive oxygen species and nitric oxide, which in turn activate other transcription factors, such as nuclear factor-κβ and activator protein-1, that enter the nucleus and cause transcription of a range of new gene products.

Preclinical studies have explored LLLT in various subsystems, including its vascular and neuroprotective functions.^10,11^ Some preclinical studies have further demonstrated improved functional recovery from TBI in animal models. One such study measured the neurologic injury severity score of mice exposed to a closed head diffuse axonal injury model of TBI.^12^ Mice were treated 4 hours after TBI with LLLT using 4 optical wavelengths: 665, 730, 810, and 980 nm. The LLLT-treated group exhibited a better functional outcome than the sham group.

Herein, we report the results of what is, to our knowledge, the first prospective, randomized, interventional clinical trial of LLLT in the setting of acute (within 72 hours) moderate TBI in humans. The study was designed to assess the safety and feasibility of applying light therapy after moderate TBI and the neuroreactivity of the injured brain to light therapy based on quantitative magnetic resonance imaging (MRI) metrics and neurocognitive function assessment. We focused the trial on moderate TBI to enable easier MRI acquisition compared with patients who experienced severe TBI and are admitted to the neurointensive care unit. In addition, this TBI category ascertained significant injury to the brain and ruled out near-normal neurologic status, as might be seen in the setting of mild TBI.

## Methods

From November 27, 2015, through July 11, 2019, we conducted a single-center, prospective, double-blind, placebo-controlled, parallel-group trial in which patients with moderate TBI were randomly assigned to LLLT or sham treatment. Patients were recruited in the emergency department of the Massachusetts General Hospital. This Health Insurance Portability and Accountability Act–compliant trial protocol was approved by the institutional review board at Massachusetts General Hospital and Human-Subject Research Protection Organization in the Department of Defense. Written informed consent was obtained. An independent steering committee, an independent data safety and monitoring board, and the ethics committee reviewed the trial regularly to assess conduct, progress, and safety. Participants received financial compensation. The trial protocol is available in Supplement 1. This study followed the Consolidated Standards of Reporting Trials (CONSORT) reporting guideline for randomized clinical trials.

**Figure 1.**
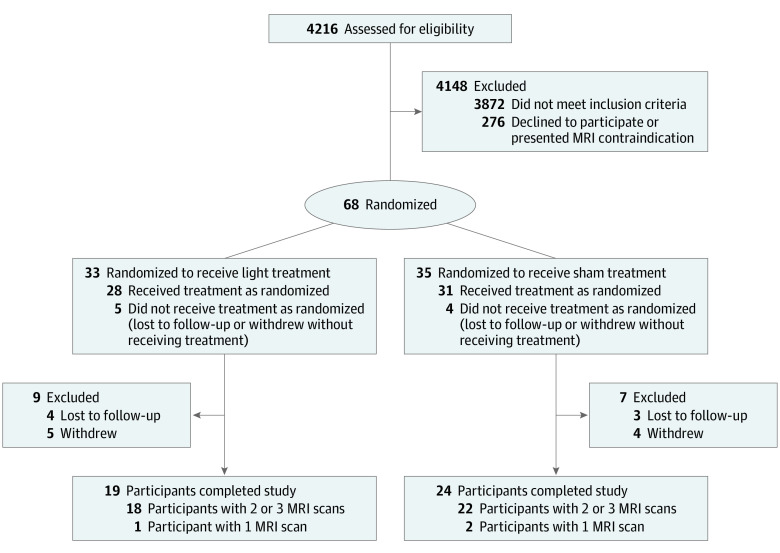
Patient Flow Diagram MRI indicates magnetic resonance imaging.

A total of 4216 men and women aged 18 to 79 years with acute, blunt TBI were screened to recruit patients with moderate TBI within 72 hours of injury. Detailed inclusion and exclusion criteria are provided in the eMethods in Supplement 2. Upon enrollment, patients were randomized using an interactive, web-based response system with a block design of 9 groups of 10 patients, each with 1:1 assignment to either the LLLT or sham group (Figure 1). The principal investigator (R.G.) and other study staff (except for the designated study coordinator [M.G.F.L.]), the patients, and the outcomes assessors were blinded to the randomization.

### Study Procedures

Low-level light therapy, which was provided by a custom-built helmet outfitted with 18 clusters of 20 NIR light-emitting diodes (the eMethods in Supplement 2), was started within 72 hours after the TBI. Treatment was divided into 3 sessions of 20 minutes’ duration with at least 12-hour intervals between the therapy sessions. The helmet provided an incident fluence of approximately 43 J/cm^2^ (0.036 W/cm^2^ × 20 minutes ×60 seconds/min = 43.2 J/cm^2^) to the scalp per 20-minute session. Based on known scalp/skull transmission of NIR light in cadavers, approximately 3% (or 1.3 J/cm^2^) of the incident fluence reached the cortical surface of the brain.

The sham treatment was delivered using the same helmet with the controller maintained on the control position during the application. This position guaranteed that the fans were switched on but the light-emitting diodes remained in the off position and did not produce any NIR light. Because the NIR light is nearly invisible to the human eye, clinical staff members in the room and participants were not able to detect whether light therapy was being given (eFigure 1 in Supplement 2).

Following the helmet application, a baseline brain MRI scan was performed for evaluation of the acute stage of the trauma. The images were acquired at the earliest opportunity, as soon as the patient was clinically stable to undergo the MRI scan (the eMethods in Supplement 2). Subsequently, we acquired 2 follow-up scans, 1 in the early (approximately 2-3 weeks after the trauma) and 1 in the late (approximately 3 months after the trauma) subacute stages of recovery. When an MRI scan could not be acquired on time because of any reason, it was performed as soon as feasible.

Detailed methods for assessing structural data are provided in the eMethods in Supplement 2. Briefly, we used brain imaging software (FreeSurfer; FreeSurfer Inc) to perform automated segmentation and cortical parcellation of the T1-weighted volumetric images.^13^ The presence of chronic white matter disease was evaluated using Fazekas scale, which is used to quantify chronic small-vessel ischemia disease based on T2 hyperintensity.^14^ The scale is split into periventricular and deep white matter, and the score ranges from 0 (no disease) to 3 (the most severe disease). A neuroradiologist (R.G.) evaluated the 3D T2-SPACE-FLAIR images to detect the presence of T2 hyperintensities and their degree (0, absent; 1, mild; 2, moderate; and 3, severe). The longitudinal stream from the software Tracts Constrained by Underlying Anatomy (TRACULA)^15^ was used to automatically delineate 18 major white matter tracts in each participant.

For each white matter tract, 2 main diffusion parameters, namely, radial diffusivity (RD) and axial diffusivity (AD), were calculated. Radial diffusivity, in part, reflects the overall integrity of myelination in a tract (water diffuses more through demyelinated axonal membranes), while AD is a measure of overall axonal integrity (water diffuses less along sheared axons).^16,17^ From the RD and AD values, which represent 2 independent parameters for each tract, 2 derived parameters—fractional anisotropy (FA) and mean diffusivity (MD)—were also calculated to obtain a measure of the overall health of a white matter tract.

Clinical assessments were performed using the Rivermead Post-Concussion Symptoms Questionnaire (RPQ)—a validated questionnaire to access postconcussion syndrome^18,19^—at baseline, 14 days, 3 months, and 6 months after the trauma. The RPQ is a 16-item self-assessment questionnaire completed via an in-person or phone interview. Each item in the questionnaire is assessed on a 5-point scale ranging from 0 (no problem) to 4 (severe problem). The questions in the RPQ can be grouped in 2 nonoverlapping sets: the RPQ-3 includes early, objective, physical symptoms, and the RPQ-13 group includes later, more cognitive and behavioral symptoms. The RPQ-3 encompasses headache, dizziness, nausea, and vomiting. The RPQ-13 includes questions evaluating noise sensitivity, sleep disturbance, fatigue, irritability, depressed mood, forgetfulness, poor concentration, longer thinking time, blurred vision, light sensitivity, double vision, and restlessness. The RPQ-3 score ranges from 0 to 12 (best to worst) and the RPQ-13 ranges from 0 to 52 (best to worst). The baseline RPQ score was assessed in the emergency department at the first opportunity when the patient became capable of answering the questions after their enrollment into the study.^20^ Clinical data were collected through an electronic data-capture system with built-in checks to assess data integrity and flag missing values.

### Outcome Measures and Hypotheses

The outcome measures were safety profile of LLLT compared with the sham treatment and the effect of LLLT on diffusion parameters of the 18 major white matter tracts and the RPQ scores compared with the sham treatment. The null hypothesis was that there was no difference between the LLLT and sham treatment groups in terms of adverse events (safety), RPQ scores (clinical symptoms of TBI), and diffusion parameters (neuroreactivity).

Instead of using a more sophisticated neuropsychological test instrument, our study relied on the RPQ score. All patients in this study were enrolled in the emergency department, and the patient enrollment spanned more than 2.5 years. the specifics of the study design necessitated that the baseline neuropsychological test be administered in the emergency department. The RPQ is feasible for this setting: it is a rapid questionnaire, making it appropriate for use in the emergency department environment. The RPQ can be administered by any trained researcher at any time of the day, avoiding the necessity of a higher-level specialist to be available in the emergency department at all times. The RPQ also has good test-retest reliability.^20^ The study was powered to show neuroreactivity using quantitative MRI metrics. Specifically, the study was not powered to show statistically significant differences on clinical outcome measures or any neuropsychological parameters. The variability of clinical and/or behavioral measures would have necessitated a much larger patient enrollment.

### Statistical Analysis

All statistical analyses were performed using R, version 3.6.0 (The R Foundation for Statistical Computing). Differences and associations were considered statistically significant at a 2-tailed *P* < .05. All results are presented as mean (SD) unless noted otherwise. Baseline differences across groups were compared using 1-way analysis of variance for continuous variables (eg, age and RPQ scores) and via χ^2^ test for categorical variables.

To test neuroreactivity of LLLT via its effect on the diffusion tensor parameters, we used a linear mixed-effect (LME) model with treatment (LLLT vs sham) and time point (acute, early subacute, and late subacute) as fixed effects and tract and time point nested within each patient as random effects. All patients with at least 1 MRI were included in this analysis. Conformity of the data to statistical assumptions was checked by investigating QQ plots of the data. Linear mixed-effect models, which are akin to multiple regression models that can account for correlations due to repeated measures, have several advantages in this context. Linear mixed-effect modeling allowed us to pool all of the data across treatment groups and 3 time points, as well as across all 18 tracts, to examine associations robustly. Moreover, because the LME model allows partially repeated measures, it can accommodate data from patients with missing time points (eg, missing MRI sequences). In addition, the LME model treats each time point as a separate variable. Therefore, any baseline difference in diffusion tensor imaging parameters across the LLLT and sham groups did not confound the overall analysis, enabling one to robustly identify differences in diffusion tensor imaging parameters across the treatment groups while explicitly accounting for repeated measurements, within-tract and within-patient correlations, and missing time points.

A similar LME model (treatment group and time point as fixed effects, and patient as a random effect) was used to test for the effect of LLLT on clinical symptoms). The *P* values for the fixed effects in the LME model were calculated using a type III analysis of variance table with the Satterthwaite degrees of freedom method.^21^

## Results

Of the 4216 patients screened, 344 patients met the inclusion/exclusion criteria and 68 were enrolled in the study between 2015 and 2018. Nine patients discontinued the study before any study-specific procedures were performed because they either withdrew consent (n = 5) or were lost to follow-up (n = 4) before their first LLLT session. The remaining 59 patients were assigned to LLLT or sham treatment groups. An additional 16 patients were lost to follow-up or withdrew consent before the first MRI scan. Forty-three patients (21 [48.8%] women, 22 [51.2%] men; mean [SD] age, 50.5 [17.4] years) had at least 1 MRI scan, and 40 patients completed MRI scans at least at 2 points: 18 in the LLLT group and 22 in the sham group (Figure 1).

All of the 68 included patients were selected based on abnormal findings from head computed tomographic imaging. Their Glasgow Coma Scale score at hospital admission ranged between 13 and 15, except for 1 patient (1.5%) who was intubated before arriving at the hospital, and the Glasgow Coma Scale score was assessed after extubation. Twelve patients (17.6%) were under the influence of alcohol or drugs at the time of evaluation, which could be a confounder for the Glasgow Coma Scale evaluation. During the course of hospitalization, 6 patients (8.8%) needed intensive care unit support and 32 patients (47.1%) needed physical or occupational therapy (Table 1). All patients received whatever supportive treatment was needed for their TBI and other associated injuries following the hospital guidelines and the judgment of their care team. Thirty-eight patients (55.9%) received antiepileptic drug treatment during the first 7 days for seizure prophylaxis (Table 1; eTable 1 in Supplement 2). For the 43 patients included in our analysis, there were no substantial differences between the groups at baseline in terms of demographics (age and sex), nature of injury, or clinical findings (Table 1; eTable 1 in Supplement 2).

**Table 1.** Demographic and Clinical Characteristics of 43 Participants Who Completed the Study

Parameter	No. (%)			*P* value
	Total (N = 43)	Sham (n = 24)	LLLT (n = 19)	
Sex				
Women	21 (48.8)	12 (50.0)	9 (47.4)	.864
Men	22 (51.2)	12 (50.0)	10 (52.6)	
Age, mean (SD), y	50.49 (17.44)	54.00 (14.68)	46.05 (19.93)	.14
Injury mechanism				
Bike/motorcycle accident				.10
With helmet	4 (9.3)	4 (16.7)	0	
Without helmet	1 (2.3)	0	1 (5.3)	
Fall	25 (58.1)	14 (58.3)	11 (57.9)	
Other	1 (2.3)	1 (4.2)	0	
Pedestrian accident with car/motorcycle/bike	4 (9.3)	3 (12.5)	1 (5.3)	
Car crash				
Restrained	3 (7.0)	0	3 (15.8)	
Unrestrained	1 (2.3)	1 (4.2)	0	
Violence/assault	4 (9.3)	1 (4.2)	3 (15.8)	
History				
Hypertension	15 (34.9)	9 (37.5)	6 (31.6)	.69
Diabetes (type 1 or 2)	7 (16.3)	4 (16.7)	3 (15.8)	.94
Imaging findings				
Hemorrhage				
Extracranial	18 (41.9)	10 (41.7)	8 (42.1)	.98
Epidural	2 (4.7)	0	2 (10.5)	.10
Acute subdural	13 (30.2)	9 (37.5)	4 (21.1)	.24
Subarachnoid	19 (44.2)	13 (54.2)	6 (31.6)	.14
Edema	1 (2.3)	1 (4.2)	0 (0.0)	.37
Contusion	4 (9.3)	2 (8.3)	2 (10.5)	.81
Hemorrhage				
Intraparenchymal	7 (16.3)	3 (12.5)	4 (21.1)	.45
Intraventricular	1 (2.3)	0 (0.0)	1 (5.3)	.26
Skull fracture	10 (23.3)	6 (25.0)	4 (21.1)	.76
Intracranial air	2 (4.7)	1 (4.2)	1 (5.3)	.87
Facial fracture	12 (27.9)	8 (33.3)	4 (21.1)	.38
Orbital injury	5 (11.6)	3 (12.5)	2 (10.5)	.84
Fazekas scale^a^				
Periventricular white matter hyperdensities				
Absent (0) / caps or pencil-thin lining (1)	37 (86.0)	19 (79.2)	18 (94.7)	.44
Smooth halo (2)	4 (9.3)	3 (12.5)	1 (5.3)	
Irregular periventricular signal extending into the deep white matter (3)	1 (2.3)	1 (4.2)	0	
Deep white matter hyperdensities				
Absent (0) / punctate foci (1)	35 (81.4)	19 (79.2)	16 (84.2)	.64
Beginning confluence (2)	6 (14.0)	3 (12.5)	3 (15.8)	
Large confluent areas (3)	1 (2.3)	1 (4.2)	0	
Antiepileptic drug prophylaxis	24 (55.8)	14 (58.3)	10 (52.6)	.71
Therapy				
Physical	19 (44.2)	13 (54.2)	6 (31.6)	.14
Occupational	13 (30.2)	8 (33.3)	5 (26.3)	.62
Speech	5 (11.6)	4 (16.7)	1 (5.3)	.25
Rehabilitation	3 (7.0)	3 (12.5)	0	.11
ICU stay	3 (7.0)	3 (12.5)	0	.11
RPQ scores, mean (SD)^b^				
RPQ-3	4.28 (3.13)	4.55 (3.13)	3.94 (3.19)	.56
RPQ-13	11.92 (8.71)	12.64 (7.83)	10.94 (9.98)	.56
RPQ-Total	16.21 (10.83)	17.18 (10.01)	14.88 (12.08)	.53

Abbreviations: LLLT, low-level light therapy; RPQ, Rivermead Post-Concussion Questionnaire.

^a^ The Fazekas scale is split into periventricular and deep white matter, and the score ranges from 0 (no disease) to 3 (the most severe disease).

^b^ The RPQ is a 16-item self-assessment questionnaire. Each item in the questionnaire is assessed on a 5-point scale ranging from 0 (no problem) to 4 (severe problem). RPQ-3 assessment includes early, objective, and physical symptoms of TBI; RPQ-13 assessment includes later, more cognitive and behavioral symptoms.

### Feasibility and Safety

In this study, 28 patients received LLLT. We were able to obtain conclusive, self-reported safety data via interviews from 19 of these patients and no adverse events referable to LLLT were identified. For the remaining 9 patients, direct phone calls or other methods to contact them failed. We reviewed their medical records and primary care physician notes for any reported adverse events or hospital admissions. No such adverse events were identified by this review of records. We, therefore, assumed them to be free of any adverse events referable to LLLT.

All 18 patients who successfully completed LLLT were able to do so without any complications. The helmet application was feasible even in patients with subgaleal hemorrhage, soft tissue swelling, and small dressings applied to portions of the head. Some patients found the helmet to be tight and noted that they would have preferred a slightly bigger helmet. There were no adverse events associated with the helmet application in either group. The symptoms described by the patients during their RPQ interview were all common symptoms of TBI.

**Figure 2.**
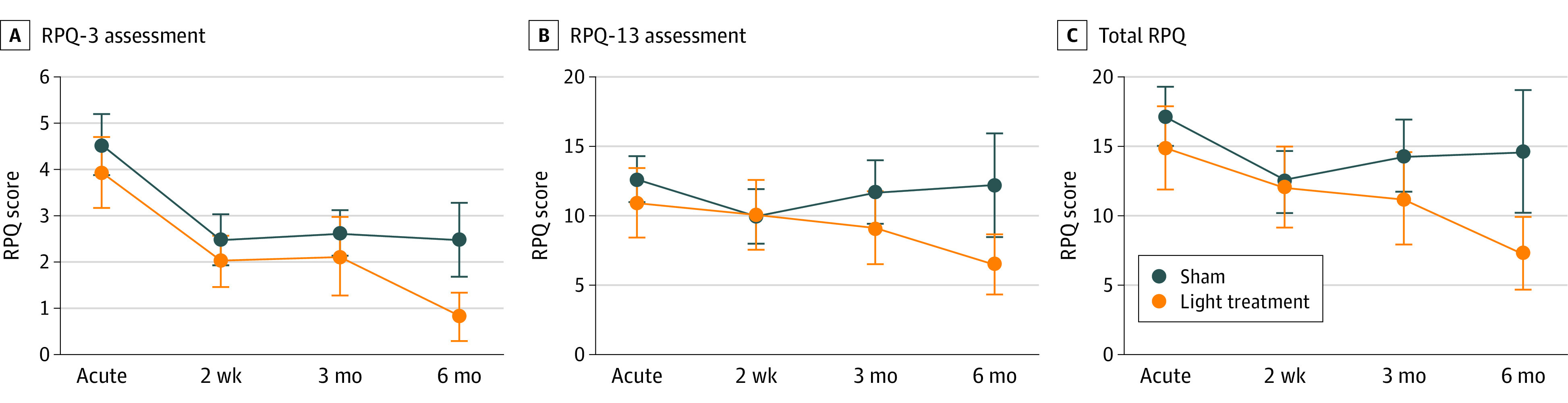
Evolution of Clinical Symptoms of Traumatic Brain Injury (TBI) in the Low-Level Light Therapy and Sham Groups Scores on the Rivermead Post-Concussion Symptoms Questionnaire, a 16-item self-assessment questionnaire. Each item in the questionnaire is assessed on a 5-point scale ranging from 0 (no problem) to 4 (severe problem). Bars show the standard error of the mean. A, Scores from RPQ-3 assessment, including early, objective, and physical symptoms of TBI. Time: *P* < .001, treatment: *P* = .40, and time × treatment: *P* = .97. B, Scores from RPQ-13 assessment, including later, more cognitive and behavioral symptoms. Time: *P* = .91, treatment: *P* = .67, and time × treatment: *P* = .89. C, Total RPQ scores. Time: *P* = .39, treatment: *P* = .61, and time × treatment: *P* = .91.

There was a progressive decrease in RPQ-3 symptom severity score—reflective of 3 objective symptoms (ie, headache, dizziness, and nausea/vomiting)—throughout the study up to the 6-month follow-up (mean [SD] sham: 4.5 [3.1] in the acute phase vs 2.5 [3.3] at 6 months, LLLT: 3.9 [3.2] vs 0.8 [1.7], and LME time effect, *P*<.01) (treatment:* P* = .40 and time × treatment: *P* = .97). Across both groups, there was no statistically significant decrease in time effect with RPQ-13 (*P* = .91) (treatment: *P* = .67, and time × treatment: *P* = .89) and RPQ-total (*P* = .39) (treatment: *P* = .61, and time × treatment: *P* = .91). Even though there was an apparent reduction in all 3 symptom scores in the LLLT group, this difference did not reach statistical significance (treatment effects *P* > .40, and time × treatment interaction *P* > .89 for all 3 RPQ measures (Figure 2). That TBI symptom severity was comparable across the LLLT and sham groups over time suggests the safety of LLLT in the acute stage of trauma.

### Neuroreactivity

One MRI scan was deemed low quality and excluded from analysis; for the remaining 42 patients (18 in the LLLT group and 24 in the sham group), there was at least 1 MRI scan available for quantitative analysis. A summary of all diffusion parameters is presented in eTable 2 in Supplement 2.

**Figure 3.**
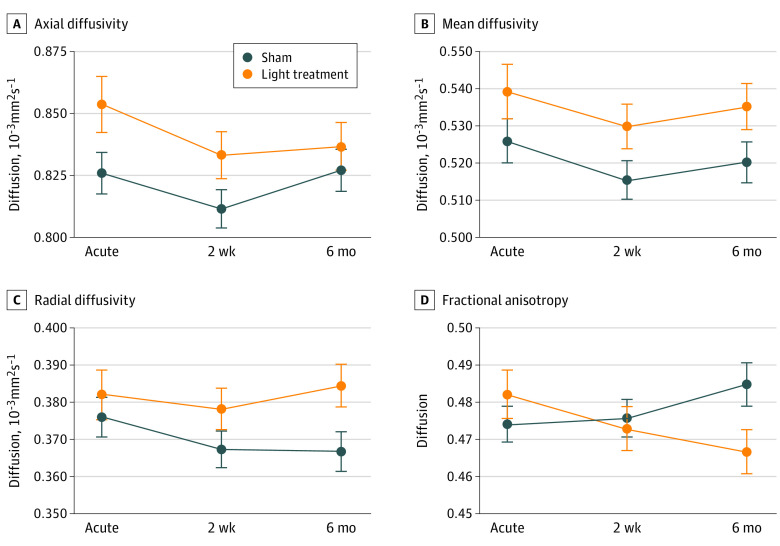
Effect of Light Treatment on Diffusion Parameters The effect of light treatment on diffusion parameters as predicted by linear mixed effect model. Error bars represent standard error of the mean. A, Axial diffusivity. Time, *P* = .02; treatment, *P* = .89; time x treatment, *P* = .47. B. Mean diffusivity. Time, *P* = .04; treatment, *P* = .08; time x treatment, *P* < .001. C, Radial diffusivity. Time, *P* = .11; treatment, *P* = .77, time x treatment, *P* < .001. D, Fractional anisotropy. Time, *P* = .02; treatment, *P* = .58; time x treatment, *P* < .001.

Table 2 presents the point estimates for the fixed effects (time, treatment group, and their interaction) from the LME model for diffusion parameters at different stages of recovery and for different treatment groups (LLLT and sham). The variance components of the random effects are given in eTable 3 in Supplement 2. Temporal trajectories of each tract are shown in eFigure 2 in Supplement 2. Passage of time had a statistically significant effect on all diffusion parameters except RD, irrespective of light or sham treatment status. The probability that the manifested temporal evolution (Figure 3) could arise purely from chance was computed by LME model to be 0.11, 0.02, 0.04, and 0.02 for RD, AD, MD, and FA, respectively. In addition, all diffusion parameters except AD were modulated by treatment status. The probability that the exhibited interaction between time and treatment status (Figure 3) could arise purely from chance was RD (*P* < .001), AD (*P* = .47), MD (*P* < .001), and FA (*P* < .001). Our results show that the temporal evolution of all diffusion parameters (except RD) was modulated by time course, and further impacted by light treatment for all parameters except AD.

**Table 2.** Point Estimates of the Diffusion Parameters at Different Stages of Recovery and Effect of LLLT

Predictor	Axial diffusivity, 10^−3^mm^2^s^−1^		Radial diffusivity, 10^−3^mm^2^s^−1^		Mean diffusivity, 10^−3^mm^2^s^−1^		Fractional anisotropy	
	Estimate (95% CI)	*P* Value	Estimate (95% CI)	*P* Value	Estimate (95% CI)	*P* Value	Estimate (95% CI)	*P* Value
Intercept^a^	0.831 (0.771-0.892)	<.001	0.374 (0.345-0.402)	<.001	0.526 (0.487-0.565)	<.001	0.479 (0.467-0.491)	<.001
Early subacute stage	−0.003 (−0.011 to 0.005)	.44	0.004 (−0.000 to 0.008)	.055	0.002 (−0.003 to 0.006)	.49	−0.006 (−0.011 to −0.001)	.02
Late subacute stage	−0.009 (−0.017 to −0.001)	.04	−0.009 (−0.013 to −0.005)	<.001	−0.009 (−0.013 to −0.004)	<.001	0.005 (0.000-0.010)	.04
LLLT	0.009 (−0.083 to 0.102)	.85	0.004 (−0.040 to 0.048)	.84	0.006 (−0.053 to 0.065)	.84	0.001 (−0.017 to 0.019)	.95
Early subacute stage × treatment	−0.007 (−0.020 to 0.006)	.27	−0.006 (−0.012 to 0.000)	.06	−0.007 (−0.014 to 0.001)	.08	0.001 (−0.007 to 0.009)	.77
Late subacute stage × treatment	−0.001 (−0.014 to 0.012)	.84	0.013 (0.006-0.019)	<.001	0.008 (0.001-0.015)	.03	−0.018 (−0.026 to −0.010)	<.001

Abbreviation: LLLT, low-level light therapy.

^a^ The estimated mean value at the acute stage across both groups. Estimates for early and late subacute stage show the predicted difference from the acute stage (intercept) across both groups, and the estimate for LLLT shows the predicted difference between the treatment groups across all time points. The last 2 predictors show the interaction between recovery stages (early vs late subacute) and treatment (light vs sham) groups.

In both groups, a time-dependent evolution of all diffusion parameters (AD, RD, MD, and FA) was evident even at the subacute stage for FA (−0.006, 95% CI, −0.011 to −0.001; *P* = .02), but not for RD (*P* = .06), AD (*P* = .44), or MD (*P* = .49) (Table 2; Figure 3). Time-dependent change in all 4 parameters across both groups reached statistical significance in the late subacute stage for AD (*P* = .04), RD (*P* < .001), MD (*P* < .001), and FA (*P* = .04).

When considered across all time points (ie, without taking into account any time-dependent evolution of parameters), LLLT did not have a significant effect on diffusion parameters (treatment effect *P* > .80 for all 4 parameters). However, there was a time and treatment interaction effect at the late subacute stage for RD (0.013; 95% CI, 0.006-0.019; *P* < .001), MD (0.008; 95% CI, 0.001-0.015; *P* = .03), and FA (−0.018; 95% CI, −0.026 to −0.010; *P* < .001), but not for AD (−0.001; 95% CI, −0.014 to 0.012, *P* = .84).

Accounting for sex, age, or for variable lengths of white matter tracts by normalizing diffusion tensor imaging parameters by the length of each tract did not change these results. Our results demonstrate that LLLT modulates the temporal evolution of RD, MD, and FA.

## Discussion

Consistent with our primary hypothesis, this study indicated the feasibility and safety of LLLT for patients with moderate TBI. Low-level light exposure is purported to confer beneficial vascular and neuroprotective effects. This beneficial effect is supported by multiple preclinical studies in animal models.^10,11,12,22,23,24^ The first clinical study of LLLT, which targeted ischemic stroke (NEST-1),^25^ delivered light through a handheld device placed against the shaved scalp at 20 predetermined locations for 2 minutes at each location. In NEST-1, the treated group showed improvements on the National Institutes of Health Stroke Severity scale over the study timeframe (ie, baseline and 5, 30, 60, and 90 days after stroke). A follow-up study of 660 patients (NEST-2)^26^ demonstrated the benefit of the LLLT, which was statistically significant after controlling for several confounding variables (advanced age, multiple strokes, and more severe strokes).^27^ NEST-3 was terminated after an interim analysis showed no difference in the primary end point between the transcranial laser therapy and sham groups.^28^

Despite these studies, a fundamental question about the neuroreactivity of LLLT remains. Our results provide what is, to our knowledge, the first direct evidence that trans-cranial LLLT targets and engages neural substrates that play an integral role in the pathophysiologic effects of moderate TBI.

Our observation that LLLT modulates temporal evolution of radial diffusivity supports the notion that light therapy affects myelin repair pathways. The process of demyelination/remyelination is distinct from axonal damage to a neuron. Therefore, changes in RD and AD can be decoupled as they are governed by distinct pathophysiologic mechanisms. Because the temporal evolution of RD, but not AD, is statistically significantly affected by light therapy, our results suggest that these 2 processes are differentially affected by NIR light for moderate TBI. Since the degree of damage to the axons and surrounding myelin is a function of the severity of the neurotrauma, our observations should not be generalized to mild or severe TBI.

The decrease in FA at the late subacute phase could be interpreted as an indication that NIR aggravates or amplifies the injury. However, the clinical evolution (Figure 2) does not support this since the RPQ scores at 6 months were lower in the LLLT group. Moreover, the total NIR light dose deployed in this study is less than the established threshold dose when light may cause cell retardation and potential neural damage.^29^ A prior animal model of myelin remodeling has shown a U-shaped evolution of the FA and RD, where a decrease in FA and an increase in RD precedes an increase in FA and a decrease in RD.^30^ That is, neurons pass through intense demyelination and remyelination processes over the recovery period. Therefore, it is possible that our late subacute phase (3 months) may coincide with the period during which initial decrease in FA (ie, early demyelination) is evident, and that our imaging timeline may not reflect the whole evolution of remyelination.

### Limitations

The study has limitations. First, our study was not designed to explore the physiologic underpinnings of the observed light-mediated biomodulation. It was also not sufficiently powered to assess the efficacy of LLLT in improving the symptom burden after moderate TBI. We studied a relatively small number of patients, partly because moderate TBI is an uncommon trauma category: less than 10% of the screened individuals during the recruitment period were eligible for our study. The relatively modest sample size in our study does not permit one to definitively conclude that LLLT will produce any clinically significant effects. The logical next steps after establishing feasibility, safety, and neuroreactivity—outcomes that our study prove—are to understand the pathophysiologic basis for neuroreactivity and study the effect of LLLT on clinical outcome measures using a larger, possibly multicenter, clinical trial.

To guide any future studies, it is instructive to estimate the sample size required to demonstrate the effect of LLLT on the RPQ score. Using the current data, we calculated the required sample size to detect a statistically significant difference in 6-month RPQ scores between the sham vs LLLT groups. We will need data from 50 patients in each group to detect a statistically significant difference in RPQ-13 score and 70 patients to detect a difference in the total RPQ score, with 0.80 statistical power.

Another limitation of our study is the lack of 6- or 12-month follow-up MRI scans. In retrospect, such a long-term follow-up of an acute intervention would have been revealing as it would have provided more information regarding the evolution of diffusion parameters and the ability of LLLT in modulating the parameters over an extended period of time. Edlow et al^31^ reported a complex longitudinal association between diffusion parameters and TBI outcome: depending on the tract evaluated, higher or lower values can estimate better or worse outcomes in the acute or subacute phase (defined as 13-97 days) of the trauma.

## Conclusions

Taken together, studies to date reinforce the need for long-term follow-up, and future multicenter clinical trials are warranted to quantitatively assess the efficacy of NIR LLLT for alleviating the symptoms of TBI. Our results show the feasibility and safety of conducting such a trial and provide a rationale for it.

## Notes

Supplement 1. **Trial Protocol**

Supplement 2. **eMethods.** Detailed Methods **eReferences** **eTable 1.** Demographic and Clinical Characteristics of Participants at Enrollment Into the Study **eTable 2.** Summary of the Brain Diffusion Parameters Based on the Time-Point and Treatment Group **eTable 3.** Random Effects and Other Statistical Parameters Derived From the Linear Mixed Effect Model **eFigure 1.** LLLT Helmet **eFigure 2.** Temporal Evolution of Each of the 18 Individual White Matter Tracts

Supplement 3. **Data Sharing Statement**
