# Supplementary material for: Effect of Transcranial Low-Level Light Therapy vs Sham Therapy Among Patients With Moderate Traumatic Brain Injury: A Randomized Clinical Trial
Source: JAMA Netw Open. 2020 Sep 14;3(9):e2017337. doi: 10.1001/jamanetworkopen.2020.17337 (PMC7490644; doi:10.1001/jamanetworkopen.2020.17337)
Supplement: Supplement 1. — Trial Protocol [file jamanetwopen-e2017337-s001.pdf]

Low-level light therapy (LLLT) with near infrared light emitting diodes in patients with moderate traumatic brain injury

**Protocol Number:**	2013P000430
**Principal Investigator:**	Rajiv Gupta, M.D., PhD<br>Associate Radiologist, MGH<br>Director, Advanced X-ray Imaging Sciences (AXIS) Center<br>Assistant Professor, Harvard Medical School<br><br>Radiological Associates<br>55 Fruit Street<br>Boston, MA 02114-2696<br><br>Phone: 617-726-0369<br>Email: RGUPTA1@PARTNERS.ORG
**Date of Issue:**	07.13.2018

Protocol Synopsis

Low-level light therapy (LLLT) with near infrared light emitting diodes in patients with moderate traumatic brain injury

**Trial Population**	Males and females 18 years and older admitted to the Emergency Department (ED) or Trauma Service with a head injury requiring hospital admission as either an inpatient or for observation.
**Investigational Product**	Custom designed Photomedex helmet with LED cluster heads emitting near infrared light.
**Control**	Randomized subjects assigned to the placebo procedure (non-activated Photomedex helmet)
**Trial Design**	Double-blinded placebo controlled study
**Primary Objective**	Safety and feasibility of using the Photomedex helmet for LLLT and quantify the response to LLLT using magnetic resonance imaging and clinical outcome measures.
**Number of Subjects**	Up to 90 subjects at Massachusetts General Hospital will be enrolled into the trial.
**Key Inclusion Criteria**	• At least 18 years old;<br>• Injury within 72 hours at the time of consent;<br>• Head injury requiring hospital admission;<br>• A Glasgow Coma Scale (GCS) score of 9-12 or 13-15 with abnormal imaging.
**Key Exclusion Criteria**	• Need for emergency neurosurgical intervention (including placement of intracranial pressure monitoring devices or drainage catheters);<br>• Hemodynamic instability as determined by the clinician;<br>• History of any of the following: (i) brain tumor; (ii) prior TBI occurring within the past year and requiring hospital admission; (iii) a new diagnosis within the past year of either stroke or epilepsy; (iv) an established diagnosis of any of the following neurodegenerative diseases: Alzheimer's, Picks, Parkinson's, Lewy body dementia, Huntington's, amytropic lateral sclerosis, spinocerebellar ataxia, vascular dementia, HIZ-associated dementia, dementia due to

	metabolic causes (Addison, Cushing, hypothyroidism, renal failure, prophyrias, Wilson, mitochondrial diseases, Wernicke-Korsakoff syndrome, and dementia of unknown etiology.<br>• Pregnancy (all women of child-bearing age will need to have a negative pregnancy test prior to the start of the interventional portion of the study);<br>• Electrical implants such as cardiac pacemakers or perfusion pumps;<br>• Ferromagnetic implants such as aneurysm clips, surgical clips, prostheses, artificial hearts, valves with steel parts, metal fragments, shrapnel, tattoos near the eye, or steel implants, or any other contra-indications to MRI.<br>• Clinical determination that subject cannot undergo MRI<br>• Breastfeeding<br>• Unstable cervical fractures<br>• Scalp lacerations or surgical wounds severe enough to preclude safe application of device<br>• Unreliable to follow up
**Primary Endpoint**	Safety and feasibility of using the Photomedex helmet for LLLT and will be based on the number of subjects who successfully complete the study without adverse events significantly related to application of the device.
**Secondary Endpoint**	The secondary endpoint will be quantification of the subject response to LLLT using MRI and clinical outcome measures.
**Regulatory Status**	This trial will be conducted as a Non-Significant Risk device trial in accordance with both the Food and Drug Administration's regulations1 and guidelines[2].<br><br>[1] 21 CFR 812.2(b)(1)<br>[2] U.S. Department of Health and Human Services, Food and Drug Administration, Center for Device and Radiological Health (January 2006). *Information Sheet Guidance For IRBs, Clinical Investigators, and Sponsors: Significant Risk and Non-significant Risk Medical Device Studies.*

Table of Contents

Protocol Synopsis 2

Table of Contents 4

1. Background and Significance 5
   - 1.1. Prevalence, Burden, and Treatment of Traumatic Brain Injury (TBI) 5
   - 1.2. Low-level laser therapy for TBI 5
   - 1.3. Clinical evidence of efficacy and safety supporting a pilot clinical study 6
2. Specific Aims 6
3. Subject Selection 7
4. Recruitment Procedures 8
5. Consent Procedures 9
6. Investigational Device Information 11
   - 6.1. Helmet Design 11
   - 6.2. Helmet Specifications and Use 13
7. Research Design and Methods 13
   - 7.1. LLLT Photomedex Helmet Device Application 14
   - 7.2. Magnetic Resonance Imaging 15
   - 7.3. Follow Up 16
8. Concomitant Medication 16
9. Statistical Methods 16
10. Adverse Events 17
11. Risk Benefit Analysis 17
    - 11.1. Known and Anticipated Risks 17
    - 11.2. Risk Minimization 18
12. Monitoring 19
    - 12.1. Data and Safety Monitoring 19
    - 12.2. Monitoring and Quality Assurance 20

1. Background and Significance

1.1. *Prevalence, Burden, and Treatment of Traumatic Brain Injury (TBI).*

TBIs are common across both military and civilian populations. In military populations, blast-induced TBI has become a signature casualty of the Iraq and Afghanistan wars [1]. A study of recently deployed personnel estimated that 19% suffered a probable TBI [2]. At this rate, nearly 320,000 military personnel suffered a blast-related brain injury through 2008 [2]. In civilian populations, TBIs from automobile accidents, sports injuries, and other sources rank as one of the leading causes of emergency hospitalizations [3]. In the US, the CDC reports an annual TBI incidence of 1.7 million, with 580,000 TBI-associated deaths in the decade 1997-2007 [3]. Worldwide estimates place the number of annual deaths and hospitalizations associated with TBI at 10 million [4].

For a patient, the burden of TBI extends for months, years, and even a lifetime after the injury. Chronic cognitive deficits have been firmly associated with all severities of TBI. Often, neurocognitive function improves over the first year after injury but plateaus below pre-injury levels [5]. Psychological deficits are also associated with TBI; in a recent study of returning military personnel, 43% of patients who met a criteria for mild TBI suffered from PTSD compared with 16% with other (non-TBI) injuries [2, 6]. TBI can induce depression within months and extending to years after injury [2, 7]. As a result, suicide rates for military personnel with a documented TBI are higher than those without [8, 9]. Sleep disruptions, daytime fatigue, and irritability have all been associated with mild TBI and are sometimes grouped under the label of post-concussive syndrome [10, 11]. Neuromotor impairment is common in severe TBIs; a recent study estimated that one third of severe TBI patients retained neuromotor impairment 2 years following injury [12]. Finally, somatic co-morbidities linked to TBI include pulmonary, cardiovascular, and gastrointestinal dysfunction [2].

1.2. *Low-level laser therapy for TBI*

Low-level light therapy (LLLT), also known by low-level laser therapy, is unique among the many therapies tested clinically for TBI. Its mechanism of action is biostimulation by near-infrared (NIR) light, also known as photobiomodulation (PBM). The advantages of LLLT in the brain have been appreciated for over a decade [13]. First, the penetration (~2 cm) of NIR light allows a device placed against the scalp to deliver therapeutic levels of light to the cortex. Second, LLLT is very well tolerated; reports of adverse events in LLLT clinical and case studies are extremely rare [14]. Third, LLLT has a relative short approval pathway because it is regulated as a device that is applied noninvasively through the skin overlying the skull. Because of these unique advantages of LLLT, it is promising as a near-term solution for the immediate and unmet military and civilian need for TBI therapy.

Over the past decade, multiple studies have demonstrated that LLLT improves recovery in TBI [15-20] and stroke [21-25] models. In parallel to these preclinical efficacy studies, laboratory research has partially deciphered LLLT's mechanisms of action. It is broadly accepted that NIR light absorption within the mitochondrial respiratory chain initiates downstream changes in neuroprotection and vascular function [13].

1.3. *Clinical evidence of efficacy and safety supporting a pilot clinical study*

Positive preclinical results motivated a first clinical study of LLLT for stroke. The results of that study, NEST-1, were published in 2007 [26]. This double-blinded placebo controlled study tested the efficacy and safety of LLLT across 120 patients. Light was delivered through a handheld device placed against the shaved scalp. The device was moved to 20 predetermined locations, and held at each location for a duration of 2 minutes. The treated group received LLLT at an average of 16 hours after stroke. Patients were evaluated based on the NIH stroke severity (NIHSS) scale at baseline, +5 d, +30 d, +60 d, and +90 d after stroke. The treatment group in this study showed improved NIHSS over this time-frame ($p = 0.021$). In a follow-up study of 660 patients (NEST-2) [27], a post-hoc analysis of moderate to moderate-severe stroke subpopulation ($n = 434$ patients) showed a statistically significant NIHSS benefit ($p = 0.044$)[40]. A third study (NEST-3) targeting a moderate stroke populations is currently underway.

In addition to providing clinical evidence that transcranial LLLT is effective in treating brain trauma, the NEST-1/2 studies demonstrated a very strong safety profile for the intervention. Detailed safety analyses were performed in both studies of over 700 combined subjects and concluded that LLLT was not associated with any risks within the stroke population of the studies.

Transcranial PBM has not to date been studied as an adjunctive treatment for acute moderate TBI and we would like to conduct a pilot study to assess feasibility. We see this as the first of a series of studies to explore whether LLLT might be useful as a safe and effective treatment for moderate TBI.

2. Specific Aims

The goal of this research is to assess PBM using LLLT to improve outcomes in subjects with moderate traumatic brain injury (TBI). In this study we use the definition of TBI as described by the DoD: GCS between 9-12 or 13-15 with an abnormal head CT.

The specific aim of this study is to determine the feasibility of PBM with near infrared light emitting diodes (LEDs) using the Photomedex Helmet and to quantify the response to LLLT using magnetic resonance imaging (MRI) and clinical outcome measures. We hypothesize that

the Photomedex helmet will successfully deliver LLLT and that we will be able to quantify the response to LLLT through imaging and clinical outcome measures.

3. Subject Selection

In this study, we propose to conduct a double-blinded placebo-controlled trial and enroll 90 subjects with moderate TBI to confirm the safety and feasibility to: 1) use the Photomedex Helmet to deliver low-level laser light through the scalp/skull and 2) track the response of delivery through MRI and clinical examinations.

Only patients presenting to the Emergency Department (ED) and/or admitted to the Trauma Service at Massachusetts General Hospital (MGH) will be considered. The following are the inclusion/exclusion criteria for the study.

Table 1: Selection Criteria

**Inclusion Criteria**	I1. At least 18 years old;<br>I2. Injury within 72 hours at the time of consent;<br>I3. Head injury requiring hospital admission;;<br>I4. A Glasgow Coma Scale (GCS) score of 9-12 or 13-15 with abnormal imaging.
**Exclusion Criteria**	E1. Need for emergency neurosurgical intervention (including placement of intracranial pressure monitoring devices or drainage catheters);<br>E2. Hemodynamic instability as determined by the clinician;<br>E3. History of any of the following: (i) brain tumor; (ii) prior TBI occurring within the past year and requiring hospital admission; (iii) a new diagnosis within the past year of either stroke or epilepsy; (iv) an established diagnosis of any of the following neurodegenerative diseases: Alzheimer's, Picks, Parkinson's, Lewy body dementia, Huntington's, amytropic lateral sclerosis, spinocerebellar ataxia, vascular dementia, HIZ-associated dementia, dementia due to metabolic causes (Addison, Cushing, hypothyroidism, renal failure, prophyrias, Wilson, mitochondrial diseases, Wernicke-Korsakoff syndrome, and dementia of unknown etiology.<br>E4. Pregnancy (all women of child-bearing age will need to have a negative urine pregnancy test prior to the start of the interventional portion of the study);<br>E5. Electrical implants such as cardiac pacemakers or perfusion pumps;<br>E6. Ferromagnetic implants such as aneurysm clips, surgical clips, prostheses, artificial hearts, valves with steel parts, metal fragments, shrapnel, tattoos near the eye, or steel implants, or any other contra-indications to MRI.<br>E7. Clinical determination that subject cannot undergo MRI<br>E8. Breastfeeding<br>E9. Unstable cervical fractures

Table 1: Selection Criteria

	E10. Scalp lacerations or surgical wounds severe enough to preclude safe application of device<br>E11. Unreliable to follow up

4. Recruitment Procedures

Patients will be approached for enrollment by members of the research team, which include clinical research coordinators and research managers in addition to the PI and other study physicians. Recruitment and enrollment will likely occur in the Emergency Department as it is preferred that the study procedures begin as soon as possible after consent has been obtained. EPIC and other relevant patient databases will be used by qualified research and clinical staff (research coordinators, managers, and study physicians) to electronically identify patients who may be potential candidates for the study. The trauma alert/stat pager may also be used to identify potential subjects by qualified study staff.

We will recruit subjects with head injury according to our inclusion/exclusion criteria. Depending on the type of head injury patients may be admitted as an inpatient or admitted to the ED observation unit.

Admission as Inpatient

For patients that are admitted as an inpatient, recruitment may require interaction with a surrogate because the patient could have impaired decision-making capacity. All potential subjects are evaluated for the capacity to consent by a physician investigator on the study to determine whether or not surrogate consent is required. When a patient is found to be eligible, a member of the clinical team will first ask the surrogate or patient if they are willing to be approached about a research study. If the surrogate or patient declines, no further attempts will be made to enroll. If the surrogate or patient agrees to speak to someone about the research, a study staff member will approach him or her for discussion of the trial and informed consent, as further outlined in the consent procedures.

Admission to ED Observation Unit

For patients that are admitted to the ED observation unit, a clinician investigator on the study will evaluate the patient to determine whether or not he or she has impaired decision-making capacity. If the patient has impaired decision-making capacity, a surrogate will be approached. If the patient is not found to have impaired decision-making capacity, they will be asked if they are interested in hearing about our research study. If the surrogate/patient declines, no further

attempts will be made to enroll. If the surrogate/patient agrees to speak with someone about the research, a study staff member will approach him or her for discussion of the trial and informed consent as further outlined in the consent procedures.

Patients with a strong likelihood of non-adherence as described in the inclusion and exclusion criteria will not knowingly be enrolled/randomized.

Recruitment will not involve restrictions on sociodemographic factors including gender or ethnic characteristics. Recruitment will be devoid of any procedures which could be construed as coercive. As such, no members of the treating Emergency Medicine teams will be involved in obtaining consent.

5. Consent Procedures

Consent will be obtained by one of the licensed physician investigators listed on the study.

The subjects to be enrolled in this study will have confirmed traumatic brain injury (TBI), requiring hospital admission either to an inpatient floor or to ED Observation. Depending on the severity of the head injury, these subjects may have diminished capacity to provide informed consent at the time of enrollment, and therefore require a surrogate for consent. All patients will be evaluated for the capacity to consent by a physician investigator on the study to determine whether or not surrogate consent is required. If it is found that the patient does have diminished capacity, then surrogate consent will be sought by the physician investigator. Otherwise, the physician investigator will obtain written informed consent from the potential subject.

Surrogate Consent

Initial consent must be sought first from a legal guardian if available, then durable power of attorney if available, then healthcare proxy if available, or if none of these, then from a spouse or other close family member by a physician investigator. However, there may be an opportunity at a follow up time-point, when the subject can be re-consented. During the scheduled follow-up, the subject will be evaluated to determine whether or not he/she has the capacity to consent. If the subject regains the capacity to consent at the follow up they will be consented at this time. If, however, the subject still has diminished capacity but is capable of assent at the time of follow-up – assent will be obtained. Written assent is not required but any prospective subject who is generally combative or who actively protests about joining the study should not be enrolled, in spite of surrogate consent. Re-consent and/or assent at the time of follow up may be obtained by a non-physician investigator trained on the consent process.

The background of the proposed trial and the benefits and risks of the procedures and trial must be explained to the surrogate. The surrogate must sign the current IRB approved consent form prior to enrollment. This form must be presented to and signed by the surrogate as well as by the consenting investigator physician.

One of the licensed physician investigators listed on the study responsible for consent will approach the surrogate after eligibility has been determined and after the surrogate has agreed to hearing about the research study from someone other than the study staff surgeon, such as a research fellow or study nurse. Assurance that participation in this study is completely voluntary will be given. The investigator physician obtaining consent will explain in detail the protocol of the study, its purpose and potential benefits to society. The surrogate will be informed about minimal risks of routine high magnetic field, non-ionizing RF radiation involved in MR imaging and exposure to the Photomedex Helmet. The surrogate will be informed that if he or she feels uncomfortable with the study, he/she can choose to terminate the study at any time. The surrogate will be informed that their refusal to participate in the study or choosing to terminate it at some point will have no effect on care and treatment received by them now or in future. The surrogate will be informed that their personal information will be protected as per the HIPAA guidelines. Surrogates will typically have less than 12 hours to decide whether or not they wish to consider participation since we are looking to apply the light helmet as soon as possible after injury.

Prior to obtaining informed consent, information will be given in a language and at a level of complexity understandable to the surrogate in both oral and written form by the investigator or assigned designee. The surrogate will not be coerced, persuaded, or unduly influenced to participate or remain in the trial. The surrogate will be given ample time and opportunity to inquire about details of the trial and all questions about the trial should be answered to the satisfaction of the representative.

The subject and his/her surrogate will receive a copy of the signed and dated informed consent form(s).

The surrogate will also provide screening information about the subject for MR compatibility and will give written informed consent prior to the MR imaging study. Informed consent clearly states that the subject and surrogate may choose to terminate the study at any time.

Subject Consent

If the potential subject is admitted to the ED observation unit and found to have full capacity for informed consent, then surrogate consent will not be obtained. Rather, the background of the

proposed trial and the benefits and risks of the procedures and trial must be explained to the potential subject. The potential subject must sign the current IRB approved consent form prior to enrollment. This form must be presented to and signed by the potential subject as well as by the consenting investigator physician.

One of the licensed physician investigators listed on the study responsible for consent will approach the potential subject after eligibility has been determined and after the potential subject has agreed to hearing about the research study from someone other than the study staff physician, such as a research fellow or study nurse. Assurance that participation in this study is completely voluntary will be given. The investigator physician obtaining consent will explain in detail the protocol of the study, its purpose and potential benefits to society. The potential subject will be informed about minimal risks of routine high magnetic field, non-ionizing RF radiation involved in MR imaging and exposure to the Photomedex Helmet. The potential subject will be informed that if he or she feels uncomfortable with the study, he/she can choose to terminate the study at any time. The potential subject will be informed that their refusal to participate in the study or choosing to terminate it at some point will have no effect on care and treatment received by them now or in future. The potential subject will be informed that their personal information will be protected as per the HIPAA guidelines. Potential subjects will typically have less than 12 hours to decide whether or not they wish to consider participation since we are looking to apply the light helmet as soon as possible after injury.

Prior to obtaining informed consent, information will be given in a language and at a level of complexity understandable to the potential subject in both oral and written form by the investigator or assigned designee. The potential subject will not be coerced, persuaded, or unduly influenced to participate or remain in the trial. The potential subject will be given ample time and opportunity to inquire about details of the trial and all questions about the trial should be answered to the satisfaction of the representative.

The subject will receive a copy of the signed and dated informed consent form(s).

The subject will also provide screening information about the subject for MR compatibility and will give written informed consent prior to the MR imaging study. Informed consent clearly states that the subject may choose to terminate the study at any time.

6. Investigational Device Information

6.1. *Helmet Design*

To deliver LLLT acutely to a population of moderate TBI patients, we will acquire two LLLT delivery helmets that are custom manufactured with light-emitting diodes (LEDs) cluster heads arranged uniformly over the inner surface. We will work with Photomedex, Inc.

(Montgomeryville, PA) to acquire two LLLT helmet devices (one will serve as a backup device). These helmets will be modified versions of existing prototype devices Figure **1**. The Photomedex helmet will sit comfortably on the head and will emit light based energy through multiple LED cluster-heads that are contained inside the custom designed helmet.

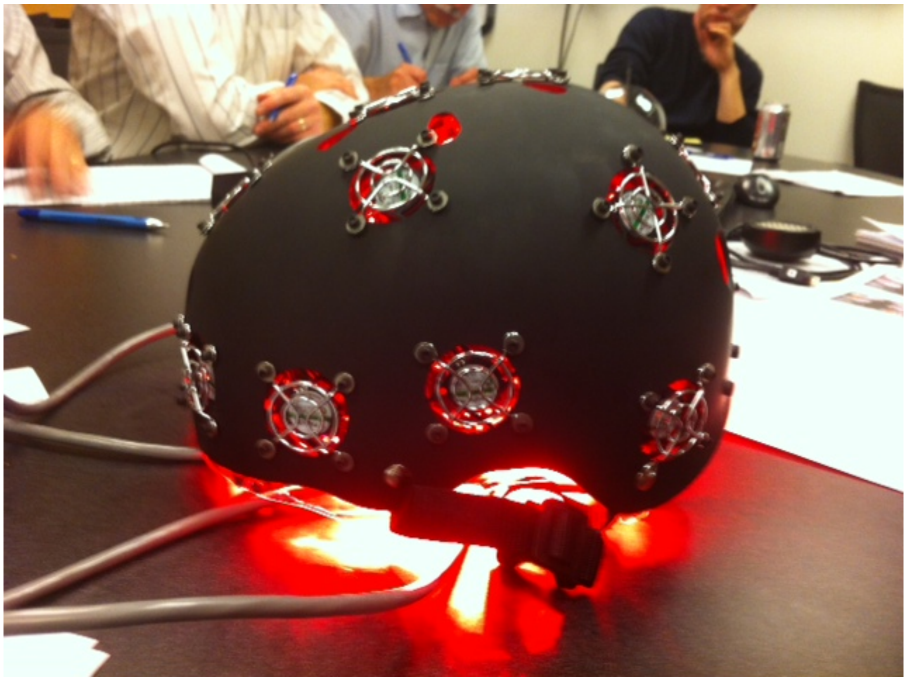

Figure 1: Photomedex Helmet Prototype

An array of 360 LED light sources will be located within the helmet. The energy that is generated by the LED sources is photothermal and therefore does not have cumulative mutagenic potential like that of ultraviolet (UV) light. Over the 20 minute procedure, this provides an incident fluence of approximately 43 J/cm2 (0.036 W/cm2 x 20 minutes x 60 seconds/minute = 43.2 J/cm2) to the scalp. Of this energy only approximately 3% or 1.3 J/cm2 will reach the cortical surface based on known scalp/skull transmission of NIR light in cadavers [28]. Heat generated by the LEDs themselves and by the control electronics will be dissipated by fans located on the exterior of the helmet (see Figure **1**). The core temperature of the subject will be monitored during exposure to ensure that there is not an increase of more than 0.5 C during the study procedure. A control unit will be connected to the helmet by a 5 ft cable, and will be used to control the helmet state (on/off/sham).

6.2. ***Helmet Specifications and Use***

The Photomedex Helmet will be used by experienced study staff members who have undergone training and it will undergo and pass initial electrical safety and quality assurance testing. In addition, the unit will be calibrated once every six months or every ten subjects, whichever happens first, to ensure the intensity does not exceed the specified intensity of 36mW/cm2 +/- 20%. This is to ensure we are within our power density specifications to maintain data quality and consistency. This testing frequency is based on our confirmation by weekly testing that the LEDs are extremely consistent and reliable in output parameters.

The exposure generated by the Photomedex Helmet is well below the accepted ANSI standard for the safe use of lasers and laser systems (ANSI Z136.1). The accepted maximum permissible exposure (MPE) for skin exposure between 10s-10,000s is 332 mW/cm2. The Photomedex Helmet will be applied for approximately 1200s (20 minutes) at a power density of 36 mW/cm2 which is approximately 10 times below the accepted standard. In addition, the fluence of the Photomedex Helmet that will reach the cortex is approximately the same as the reported fluence of the Photothera device used in the NEST-1/2 trials (1.3 J/cm2 vs. ~1J/cm2, respectively).

The LED sources will emit at a center wavelength of 810 nm with a bandwidth of 30 nm. The intensity on the scalp will be approximately 36 mW/cm2 and will be confirmed by a custom-built light monitoring tool to vary by less than 20% across the illumination field.

7. Research Design and Methods

A member of the study staff familiar with the subject population will evaluate eligibility based on the approved inclusion/exclusion criteria, using patient charts. If a subject is eligible a physician on the study staff will approach the subject and/or surrogate for informed consent (please see consent procedures for more details). Demographic and clinical data will be collected during the acute hospitalization in accordance with the common data elements proposed by the TBI Common Data Element Project [29]. A drop point for deterioration for withdrawing a subject will be a change in the GCS of greater than 2 from the previous exam.

After informed consent has been obtained, the subject will be randomized. All subjects will undergo either treatment or sham (placebo) procedures. In the sham procedure, the helmet will be placed on the patient but the LEDs will not be activated. The randomization will be set up so subjects are assigned sequentially into one of 9 groups with 10 subjects in each group. Within each group, the subject will be assigned randomly to either treatment or sham with an allocation ratio of 1:1. Once the first 10 subjects have been enrolled and have finished treatment (or sham) a DSMB (see detailed description under data safety and monitoring) will go over all of the safety data presented and make a recommendation on the continuation of the study. Subsequent DSMB

meetings will take place once per year

If the subject is admitted to the ICU, he/she, will be treated according to the ICU standard of care procedures.

Standard of care procedures include:
Monitoring of O2/CO2 levels;
Avoiding hypotension;
Maintaining temperature within normative range;
Adjusting cervical collar placement if necessary;
Repeat CT scan if necessary;
Bed placement at > 30 degrees while in the ICU
Sedation and analgesia using recommended agents

If the subject is admitted to the ED Observation Unit, he/she will be treated according to the ED standard of care procedures.

The application of the Photomedex helmet will be in addition to but will not change the standard of care.

The following table summarizes the timing and specific assessments to be conducted in this study:

ASSESSMENTS	PRE-PROCEDURE	PROCEDURE	FOLLOW UP
Eligibility Assessment	X		
Informed Consent Form[a]	X		
Demographics, including date of birth, gender, and race and ethnicity	X		
Medical History	X		
Pregnancy Test[b]	X		X
Investigational LLLT using the Photomedex Helmet		X	
Neuroimaging using Magnetic Resonance		X	X
Clinical Outcomes Assessment		X	X
AE, SAE, UADE Assessment		X	X

[a] If the subject regains capacity they will be re-consented at the time of follow up.
[b] Exclusion criteria (E4.) specific for female subjects of childbearing age.

7.1. *LLLT Photomedex Helmet Device Application*

In order to maintain blinding, a study staff member not associated with analysis of the study will create a randomization code and assign treatment via an envelopment randomization system. The study staff member in charge of applying the Photomedex helmet will open each

randomization envelop and once the clinician deems it is acceptable, subjects will undergo approximately 20 minutes of exposure to the Photomedex helmet within 72 hours after injury. If the subject is assigned to the sham group the LEDs will not be activated. Because the helmet uses NIR light that is invisible to the human eye, clinical staff within the room will not be able to detect whether the LEDs are activated.

The Photomedex helmet will be used up to 3 times for each subject. The first application will be as soon as possible after injury, within 72 hours. Follow up applications of the Photomedex helmet will be applied for up to 2 additional time points no sooner than 12 hours after the previous helmet application. If the subject is discharged from the hospital prior to the second helmet application, a follow up helmet application will be scheduled. If the subject wishes to have the option to complete in-person visits at his/her place of residence or another location, they will indicate this by signing the LLLT Out of Hospital Follow Up Consent Form at the time they enroll in the study.

7.2. ***Magnetic Resonance Imaging***

Following the 1st Photomedex helmet application, a baseline MRI will be performed, at the earliest opportunity, as soon as the subject is able to lay flat for 1 hour and the scanner is available for use. If the subject is admitted as an inpatient the scan will take place at Lunder 6 on the MGH Main Campus. If the subject is discharged from ED Observation prior to the first MRI, then the baseline MRI will take place at the Martinos Center at the Charlestown Campus. It is anticipated that the MRI will take place within 24-48h hours after enrollment and the scanning protocols themselves will take approximately 60 minutes to perform. Taking into account any necessary set up time before, during and after scanning the total time for the MRI will be approximately 60-90 minutes. If the subject is unable to undergo research-related imaging, this will be deferred until it is safe and feasible. This baseline MRI is performed following the first application of light therapy because of the need to deliver light therapy as soon as possible after injury, and the clinical challenges of performing MRI within the first 12 hours. A designated study staff member will be responsible for gathering post-procedure follow-up data on the subject. The subjects will be followed until discharge.

<u>Imaging protocol (for baseline and post-treatment imaging):</u>

Subjects will be asked to lie still in a supine position for the duration of the imaging session. Head motion will be minimized during image acquisition by the use of a dedicated head coil with holder.

The MRI protocol may include the following sequences:
1) Localizer/Auto-aligned scout
2) High resolution T2 weighted imaging

3) FLAIR (Fluid attenuated inversion recovery) imaging
4) High resolution 3D T1 weighted isotropic imaging
5) Susceptibility-weighted imaging (SWI)
6) Diffusion tensor imaging (60 encoding directions)
7) BOLD EPI functional MRI for the evaluation of resting state functional connectivity (FC) within and between motor, visual, auditory, dorsal attention, executive control, salience and default mode networks.
8) Arterial Spin Labeling (ASL) brain perfusion imaging.

No contrast agent or other pharmacological substance other than ones clinically required for the subject will be given.

7.3. *Follow Up*

Follow up visits will be coordinated by the study staff with the subject and/or surrogate. Additional MRI scans will be performed at approximately 14-21 days and 3 months after injury for image analysis (see Section 7.2 for Imaging Protocol). At day 7 a follow-up phone call will be made to check on the subject after discharge for symptoms reporting. Clinical follow-up visits will take place at approximately 14-21 days, 3 months and 6 months post-injury. The 6 month follow up can be completed in person or over the phone since it does not require in person physical evaluation. A follow-up phone call will also be performed at day 7 and at 12 months.

For subjects who are discharged from the ED's 24-hour Observation Unit, the same follow up procedures will apply. Additionally, subjects in ED Observation who only received 1 helmet application will have up to 2 additional helmet application(s) performed by study staff at a follow up appointment. Throughout the study duration, subjects may be contacted by phone, email or mail by clinical research staff for study related reasons.

8. Concomitant Medication

Each subject's concomitant medication regimen before the start of the trial is to be documented. Concomitant medications are to be recorded on the appropriate case report form (CRF).

9. Statistical Methods

In this pilot study, the study size is limited by number of recruitable patients admitted to MGH emergency department. Based on prior-year admission records, the itemized inclusion/exclusion criteria, and an assumed 80% consent rate, we estimate an average enrollment of 1 patient per week.

The sufficiency of these enrollments toward powering the primary hypothesis (DTI outcome measure) is presented below. These calculations require an assumption of the acute LLLT therapeutic effect size, which is unknown and cannot reasonably be estimated from either the preclinical data or the clinical stroke studies. Our analysis is based on an assumed effect size of

25%.

The coefficient of variation for fractional anisotropy (FA) is based on data from a cohort of 12 mild to moderate TBI patients scanned using quantitative MRI [30]. For this cohort, FA was measured in 16 regions of the brain. The coefficient of variation (CV), defined as the ratio of mean to the standard deviation, was 53.45%. However, this study included mild to moderate. With the more narrow inclusion of moderate TBI, we estimate a FA CV of 40% (based on clinical and research experience of the investigators, and knowing that the CV for healthy volunteers is 25%). The required sample size for detecting changes in FA associated with acute LLLT [31] is then 82 (41 patients in each group). We note that other diffusion parameters (mean diffusivity, axial diffusivity, and radial diffusivity) featured much lower CV, and would be sufficiently powered with this sample size.

10. Adverse Events

Adverse events will be reviewed by the Principal Investigator, Research Monitor and the DSMB and will be reported to the Human Research Committee within the required time frame and to all participating investigators according to the Partners Human Research Committee guidelines.

Underlying diseases or conditions are not reported as AEs unless there is an increase in severity or frequency during the course of the investigation. Death should not be recorded as an adverse event, but should only be reflected as an outcome to the applicable AE that led to the death.

11. Risk Benefit Analysis

11.1. ***Known and Anticipated Risks***

We anticipate the risk to the subjects due to participation in this study to be low. Subjects who have satisfied the inclusion/exclusion criteria as outlined in the protocol will be eligible for enrollment. All subjects will be treated according to the standard of care guidelines for their condition according to the medical staff. For the experimental portion of the procedure, after consent has been obtained, subjects will undergo approximately 20 minutes of exposure to the Photomedex Helmet for at least 2 time points and up to 3 time points. The first helmet application will take place in the inpatient unit or in the ED observation unit. If the subject is admitted as an inpatient he/she may have up to 2 additional helmet applications no sooner than 12 hours after the previous helmet application. If the subject is discharged from either the inpatient unit or the ED observation unit prior to his/her 2nd helmet application, he/she will have up to 2 additional helmet applications no sooner than 12 hours after the previous helmet application arranged either through a follow up visit.

In order to optimize the LLLT, we would like to shave the head. This will be optional to the surrogate/subject and will be explained at the time of informed consent. There is a slight

potential risk of a small cut to the head during shaving of the head. In addition, there is a small amount of radiant heat that is generated by the Photomedex helmet. The helmet will be efficiently cooled with fans but depending on the skin and hair color of the subject the temperature of the scalp may be raised enough to be perceived as warmth. In addition, the subject's core temperature will be monitored and in the unlikely event that the temperature increases more than 0.5 C the helmet will be removed.

Following initial exposure to the Photomedex Helmet, a baseline MRI will be performed at the earliest opportunity as soon as the patient is able to lay flat for 1 hour and the scanner is available for scheduling. If the subject is admitted to an inpatient unit, he/she will have the first MRI scan in Lunder 6 at the MGH Main Campus. If the subject is discharged from the ED Observation unit, he/she will have the first MRI scan at the Martinos Center in Charlestown.

For subjects admitted to the inpatient unit, there is a small risk of complication associated with moving the subject from the ICU to radiology for MRI. The most common complications are related to inadvertent dislodgement of tubes and lines or related to temporary disconnection from the mechanical ventilator. These complications are inherent in any travel out of the ICU. If the risk of travel complications is deemed by the clinical (non-study) team to be too high, MRI for the purposes of this study will be deferred until a later date. The FDA considers investigations using MRI software and hardware to be non-significant risk, as long as they are operated within FDA specified parameters. All MRI studies will adhere to the FDA approved for the Siemens 3T system used in this research. These safety parameters include static magnetic field, time varying magnetic fields (dB/dt), specific absorption rate (SAR), and acoustic noise levels.

11.2. ***Risk Minimization***

This study involves the application of the Photomedex helmet for delivery of low-level laser light using LED cluster heads. The risks to the subject have been minimized by choosing an exposure that has been shown to be effective but does not exceed accepted standards. The exposure generated by the Photomedex Helmet is well below the accepted ANSI standard for the safe use of lasers and laser systems [ANSI Z136.1]. The accepted maximum permissible exposure (MPE) between 10s-10,000s is 332 mW/cm2. The Photomedex helmet will be operating for approximately 1200s and has a power density of 36mW/cm2 which is approximately 10 times below the accepted standard. In addition, the output fluence of our device per treatment session (43 J/cm2) is at approximately half of that used in the NEST-1 trial (82 J/cm2) with the Photothera device by Lampl et al in stroke patients. In this study there were no adverse events found to be significantly related to the application of the Photothera device [26]. We will also monitor the temperature of the subjects during application of the Photomedex helmet to ensure that application of the LLLT does not increase the temperature of the subject

more than 0.5 C. If a temperature above this is reached the Photomedex helmet will be removed.

This study also involves MRI of the brain without intravenous contrast. There is no additional risk to the subject associated with these proposed MRI sequences, above those of a conventional MRI that is routinely performed on these patients for clinical workup. The FDA considers investigations using MRI software and hardware to be non-significant risk, as long as they are operated within FDA specified parameters. We will ensure all imaging will take place according to the FDA specified parameters for the Siemens 3T system.

We will employ measures throughout the course of this study to minimize risks to subjects choosing to participate. All efforts will be made to minimize potential risks by:

- Providing training on the investigational equipment prior to use
- Defining inclusion/exclusion criteria clearly to ensure only appropriate subjects are enrolled.
- Ensuring that the treatment of the subject is consistent with current medical practices.

12. Monitoring

The Principal Investigator will make necessary efforts to ensure that this trial is conducted in compliance with GCPs and all applicable regulatory requirements.

12.1. ***Data and Safety Monitoring***

The PI will be responsible for overseeing all aspects of the study including: ensuring that the study is conducted according to the IRB-approved protocol, performing monitoring and confirming compliance of the study, and protecting the rights, safety and welfare of the subjects. Should the PI at any point feel that the health and well being of the subject is compromised the study procedures will be immediately suspended.

Data will be collected by the appropriate study staff, such as research fellows from the Department of Trauma, Emergency Surgery and Surgical Critical Care. Case report forms will be kept securely in the electronic Partners Redcap Database. In addition, electronic data will be stored on a Password Protected computer system and backed up onto a secure server location. Only the PI and research team will have access to the database, and identifying information will not be released. The data will be reviewed regularly by the designated study staff and the PI will regularly review the data and findings with the study staff during a regularly held research meeting. Throughout the duration of the study, the PI will perform ongoing evaluation of subject safety data in order to identify AEs and trends as soon as possible. The PI will be responsible for determining whether the research should be altered or stopped. In addition, de identified image MRI image data will be shared with external collaborators in order to perform quantitative image

analysis through a customized computational algorithm. The data will have all identifiers removed and will be transferred to the collaborator by a study staff member.

A designated study staff member will be responsible for gathering post-procedure follow-up data on the subject. The subjects will be followed until discharge. We will also follow-up with the patient 3 and 6 months after injury. A follow up phone call will be completed at 12 months.

In order to review the data in an unblinded fashion, we will establish a DSMB of experienced specialists from Trauma, Neurology, Radiology, Rehabilitation at MGH and Spaulding, and the Wellman Center at MGH who are not conflicted or study staff members to review the safety data. The DSMB will review the data collected after the first 10 subjects have been enrolled. The DSMB will make a recommendation as to whether or not the study should continue. If the DSMB recommends continuation of the study, subsequent DSMB review of data once per year.

Additionally, John Chen MD, a neurologist at MGH and member of the DSMB will serve as an independent research monitor for the study. He will review the research protocol with the investigators, review the monitoring plan, and review study data. If the research monitor is concerned for the safety and well-being of the subjects he/she will have the authority to stop the research protocol in progress, remove individual human subjects from a research protocol, and take whatever steps are necessary to protect the safety and well-being of human subjects until the IRB can assess the monitor's assessment. The research monitor will review all unanticipated problems involving risk to subjects or others, device-related serious adverse events and all subject deaths associated with the protocol. At a minimum, the research monitor must comment on the outcomes of the event or problem and in case of a device-related serious adverse event or death, comment on the relationship to participation in the study. The research monitor must also indicate whether he/she concurs with the details of the report provided by the principal investigator. The research monitor will provide an unbiased written report Reports for events determined by either the investigator or research monitor to be possibly or definitely related to participation and reports of events resulting in death will be promptly forwarded to the USAMRMC ORP HRPO.

12.2. ***Monitoring and Quality Assurance***

Monitoring of the study data will be made to ensure that all aspects of the current, approved protocol/amendment(s) are followed. Original source documents will be reviewed for verification of data. A monitoring log will be completed for proper documentation. A device accountability will also be kept for the Photomedex Helmet used in the study.

Subject data will be collected via case report forms and will be recorded in a Partners RedCap Database designed specifically for the study. The PI will ensure the accuracy and completeness

of the recorded data and will provide his/her signature to verify. If any discrepancies are found during monitoring of the data, confirmation of the correct information will be made by the PI by signature and a note to file description will be generated.

13. References

[1] D. Warden, "Military TBI During the Iraq and Afghanistan Wars," *The Journal of Head Trauma Rehabilitation,* vol. 21, 2006.

[2] "Invisible Wounds of War," *RAND Center for Military Health Policy Research,* 2008.

[3] V. G. Coronado, L. Xu, S. V. Basavaraju, L. C. McGuire, M. M. Wald, M. D. Faul, B. R. Guzman, and J. D. Hemphill, "Surveillance for traumatic brain injury-related deaths--United States, 1997-2007.," *MMWR Surveillance summaries:Morbidity and mortality weekly report Surveillance summaries/CDC,* pp. 1-32, 2011.

[4] A. A. Hyder, C. A. Wunderlich, P. Puvanachandra, G. Gururaj, and O. C. Kobusingye, "The impact of traumatic brain injuries: A global perspective," *NeuroRehabilitation,* vol. 22, pp. 341-353, Feb 01 2007.

[5] L. Ruttan, K. Martin, A. Liu, B. Colella, and R. E. Green, "Long-Term Cognitive Outcome in Moderate to Severe Traumatic Brain Injury: A Meta-Analysis Examining Timed and Untimed Tests at 1 and 4.5 or More Years After Injury," in *Archives of physical medicine and rehabilitation*, 2008, pp. S69-S76.

[6] C. W. Hoge, D. McGurk, J. L. Thomas, A. L. Cox, C. C. Engel, and C. A. Castro, "Mild Traumatic Brain Injury in U.S. Soldiers Returning from Iraq," *New England Journal Of Medicine,* vol. 358, pp. 453-463, Feb 21 2012.

[7] M. D. Kim Edward, M. D. Lauterbach Edward, M. D. Reeve Alya, M. D. Arciniegas David, P. D. Coburn Kerry, M. D. P. D. M. Mendez, M. D. Rummans Teresa, and M. D. Coffey Edward, "Neuropsychiatric Complications of Traumatic Brain InjuryA Critical Review of the Literature (A Report by the ANPA Committee on Research)," *The Journal of Neuropsychiatry and Clinical Neurosciences,* vol. 19, pp. 106-127.

[8] G. Simpson and R. Tate, "Suicidality after traumatic brain injury: demographic, injury and clinical correlates.," *Psychol Med,* vol. 32, pp. 687-697, Jun 01 2002.

[9] G. Simpson and R. Tate, "Clinical Features of Suicide Attempts After Traumatic Brain Injury," *The Journal of Nervous and Mental Disease,* vol. 193, 2005.

[10] E. D. Bigler, "Distinguished Neuropsychologist Award Lecture 1999. The lesion(s) in traumatic brain injury: implications for clinical neuropsychology.," *Archives of clinical neuropsychology : the official journal of the National Academy of Neuropsychologists,* vol. 16, pp. 95-131, Mar 2001.

[11] E. R. Peskind, E. C. Petrie, D. J. Cross, K. Pagulayan, K. McCraw, D. Hoff, K. Hart, C.-E. Yu, M. A. Raskind, D. G. Cook, and S. Minoshima, "Cerebrocerebellar hypometabolism associated with repetitive blast exposure mild traumatic brain injury in

12 Iraq war Veterans with persistent post-concussive symptoms," *NeuroImage,* vol. 54, Supplement 1, pp. S76-S82, Feb 01 2011.

[12] W. C. Walker and T. C. Pickett, "Motor impairment after severe traumatic brain injury: A longitudinal multicenter study," *J Rehabil Res Dev,* vol. 44, pp. 975-982, 2007.

[13] H. Chung, T. Dai, S. Sharma, Y.-Y. Huang, J. Carroll, and M. Hamblin, "The Nuts and Bolts of Low-level Laser (Light) Therapy," *Annals of Biomedical Engineering,* vol. 40, pp. 516-533, Feb 01 2012.

[14] Y.-Y. Huang, A. Gupta, D. Vecchio, V. J. Bil de Arce, S.-F. Huang, W. Xuan, and M. R. Hamblin, "Transcranial low level laser (light) therapy for traumatic brain injury," *J. Biophotonics,* vol. (in press), 2012.

[15] T. Ando, W. Xuan, T. Xu, T. Dai, S. K. Sharma, G. B. Kharkwal, Y.-Y. Huang, Q. Wu, M. J. Whalen, S. Sato, M. Obara, and M. R. Hamblin, "Comparison of therapeutic effects between pulsed and continuous wave 810-nm wavelength laser irradiation for traumatic brain injury in mice.," *PLoS ONE,* vol. 6, p. e26212, 2011.

[16] J. Khuman, J. Zhang, J. Park, J. D. Carroll, C. Donahue, and M. J. Whalen, "Low-level laser light therapy improves cognitive deficits and inhibits microglial activation after controlled cortical impact in mice.," *Journal of Neurotrauma,* vol. 29, pp. 408-417, Feb 20 2012.

[17] A. Oron, U. Oron, J. Streeter, L. de Taboada, A. Alexandrovich, V. Trembovler, and E. Shohami, "low-level laser therapy applied transcranially to mice following traumatic brain injury significantly reduces long-term neurological deficits.," *Journal of Neurotrauma,* vol. 24, pp. 651-656, May 2007.

[18] A. Oron, U. Oron, J. Streeter, L. de Taboada, A. Alexandrovich, V. Trembovler, and E. Shohami, "Near infrared transcranial laser therapy applied at various modes to mice following traumatic brain injury significantly reduces long-term neurological deficits.," *Journal of Neurotrauma,* vol. 29, pp. 401-407, Feb 20 2012.

[19] B. J. Quirk, M. Torbey, E. Buchmann, S. Verma, and H. T. Whelan, "Near-infrared photobiomodulation in an animal model of traumatic brain injury: improvements at the behavioral and biochemical levels.," *Photomedicine and Laser Surgery,* vol. 30, pp. 523-529, Sep 2012.

[20] Q. Wu, W. Xuan, T. Ando, T. Xu, L. Huang, Y.-Y. Huang, T. Dai, S. Dhital, S. K. Sharma, M. J. Whalen, and M. R. Hamblin, "Low-Level Laser Therapy for Closed-Head Traumatic Brain Injury in Mice: Effect of Different Wavelengths," *Lasers Surg. Med,* vol. 44, pp. 218-226, Feb 24 2012.

[21] L. DeTaboada, S. Ilic, S. Leichliter-Martha, U. Oron, A. Oron, and J. Streeter, "Transcranial application of low-energy laser irradiation improves neurological deficits in rats following acute stroke," *Lasers Surg. Med,* vol. 38, pp. 70-73, 2006.

[22] P. A. Lapchak, K. F. Salgado, C. H. Chao, and J. A. Zivin, "Transcranial near-infrared light therapy improves motor function following embolic strokes in rabbits: An extended

therapeutic window study using continuous and pulse frequency delivery modes," *Neuroscience,* vol. 148, pp. 907-914.

[23] P. A. Lapchak, J. Wei, and J. A. Zivin, "Transcranial Infrared Laser Therapy Improves Clinical Rating Scores After Embolic Strokes in Rabbits," *Stroke,* vol. 35, pp. 1985-1988, Feb 01 2004.

[24] M. C. P. Leung, S. C. L. Lo, F. K. W. Siu, and K. F. So, "Treatment of experimentally induced transient cerebral ischemia with low energy laser inhibits nitric oxide synthase activity and up-regulates the expression of transforming growth factor-beta 1," *Lasers Surg. Med,* vol. 31, pp. 283-288, Sep 25 2002.

[25] A. Oron, U. Oron, J. Chen, A. Eilam, C. Zhang, M. Sadeh, Y. Lampl, J. Streeter, L. DeTaboada, and M. Chopp, "Low-Level Laser Therapy Applied Transcranially to Rats After Induction of Stroke Significantly Reduces Long-Term Neurological Deficits," *Stroke,* vol. 37, pp. 2620-2624, Feb 01 2006.

[26] Y. Lampl, J. A. Zivin, M. Fisher, R. Lew, L. Welin, B. Dahlof, P. Borenstein, B. Andersson, J. Perez, C. Caparo, S. Ilic, and U. Oron, "Infrared Laser Therapy for Ischemic Stroke: A New Treatment Strategy," *Stroke,* vol. 38, pp. 1843-1849, Feb 01 2007.

[27] J. A. Zivin, G. W. Albers, N. Bornstein, T. Chippendale, B. Dahlof, T. Devlin, M. Fisher, W. Hacke, W. Holt, S. Ilic, S. Kasner, R. Lew, M. Nash, J. Perez, M. Rymer, P. Schellinger, D. Schneider, S. Schwab, R. Veltkamp, M. Walker, J. Streeter, and f. t. N.-. Investigators, "Effectiveness and Safety of Transcranial Laser Therapy for Acute Ischemic Stroke," *Stroke,* vol. 40, pp. 1359-1364, Feb 01 2009.

[28] S. S. Wan, J. A. J. Parrish, R. R. R. Anderson, and M. M. Madden, "Transmittance of nonionizing radiation in human tissues.," *Photochemistry and Photobiology,* vol. 34, pp. 679-681, Dec 01 1981.

[29] A. I. Maas, C. L. Harrison-Felix, D. Menon, P. D. Adelson, T. Balkin, R. Bullock, D. C. Engel, W. Gordon, J. Langlois Orman, H. L. Lew, C. Robertson, N. Temkin, A. Valadka, M. Verfaellie, M. Wainwright, D. W. Wright, and K. Schwab, "Common Data Elements for Traumatic Brain Injury: Recommendations From the Interagency Working Group on Demographics and Clinical Assessment," in *Archives of physical medicine and rehabilitation*, 2010, pp. 1641-1649.

[30] M. Singh, J. Jeong, D. Hwang, W. Sungkarat, and P. Gruen, "Novel diffusion tensor imaging methodology to detect and quantify injured regions and affected brain pathways in traumatic brain injury.," *Magnetic resonance imaging,* vol. 28, pp. 22-40, Feb 2010.

[31] G. van BELLE, "Statistical rules of thumb (Series in probability and statistics)," *Recherche,* 2008.
