# Supplementary material for: Effect of Transcranial Low-Level Light Therapy vs Sham Therapy Among Patients With Moderate Traumatic Brain Injury: A Randomized Clinical Trial
Source: JAMA Netw Open. 2020 Sep 14;3(9):e2017337. doi: 10.1001/jamanetworkopen.2020.17337 (PMC7490644; doi:10.1001/jamanetworkopen.2020.17337)
Supplement: Supplement 2. — eMethods. Detailed Methods eReferences eTable 1. Demographic and Clinical Characteristics of Participants at Enrollment Into the Study eTable 2. Summary of the Brain Diffusion Parameters Based on the Time-Point and Treatment Group eTable 3. Random Effects and Other Statistical Parameters Derived From the Linear Mixed Effect Model eFigure 1. LLLT Helmet eFigure 2. Temporal Evolution of Each of the 18 Individual White Matter Tracts [file jamanetwopen-e2017337-s002.pdf]

Supplementary Online Content

Figueiro Longo MG, Tan CO, Chan ST, et al. Effect of transcranial low-level light therapy vs sham therapy among patients with moderate traumatic brain injury: a randomized clinical trial. *JAMA Netw Open*. 2020;3(9):e2017337. doi:10.1001/jamanetworkopen.2020.17337

eMethods. Detailed Methods

eReferences

eTable 1. Demographic and Clinical Characteristics of Participants at Enrollment Into the Study

eTable 2. Summary of the Brain Diffusion Parameters Based on the Time-Point and Treatment Group

eTable 3. Random Effects and Other Statistical Parameters Derived From the Linear Mixed Effect Model

eFigure 1. LLLT Helmet

eFigure 2. Temporal Evolution of Each of the 18 Individual White Matter Tracts

This supplementary material has been provided by the authors to give readers additional information about their work.

© 2020 Figueiro Longo MG et al. *JAMA Network Open.*

eMethods. Detailed Methods

Participants

Moderate TBI was defined according to the DoD definition as: (1) Glasgow Coma Scale (GCS) score between 9 and 12, or (2) GCS between 13 and 15 with concomitant abnormal imaging findings on head computed tomography (CT), such as hemorrhagic contusions, acute intracranial hemorrhage, skull or facial fractures, or subgaleal hematoma. Eligibility was determined based on the head CT report issued by a board-certified neuroradiologist in the ED.

Patients were excluded if they presented any hemodynamic instability, needed an emergency neurosurgical intervention, had any evidence of unstable cervical fracture, suffered scalp lacerations severe enough to preclude safe application of the LLLT helmet, or were pregnant or breastfeeding. Additionally, patients were excluded if they had a history of any of the following: brain tumor, prior TBI within the past year requiring hospital admission, a new diagnosis within the past year of stroke, epilepsy, or any clinical condition that would prevent the subject from MR scanning. Conditions preventing MRI scanning included presence of electrical implants such as cardiac pacemakers or perfusion pumps, any ferromagnetic implant such as MR-unsafe aneurysm clips, surgical clips, prostheses, artificial hearts, valves with steel parts, metal fragments, shrapnel, tattoos near the eye, or steel implants. Patients were also excluded if they had a clinically diagnosed neurodegenerative disease (e.g., Alzheimer's, Picks, Parkinson's, Lewy body dementia, Huntington's, amytrophic lateral sclerosis, spinocerebellar ataxia, vascular dementia, HIV-associated dementia), dementia due to metabolic causes (e.g., Addison, Cushing, hypothyroidism, renal failure, porphyrias, Wilson, mitochondrial diseases, Wernicke-Korsakoff syndrome), or dementia of unknown etiology.

Subjects with surrogate consent were re-assessed for cognitive recovery throughout the study, and those who regained adequate capacity were given the opportunity to re-consent for themselves.

Study procedures

The general medical team taking care of the patient had no interference or involvement in the trial; they provided care in accordance with the patients' clinical needs, following the routine standard of care.

Low-Level Light Therapy (LLLT) and Sham Treatment

The LEDs emit at a center wavelength of 810 nm with a bandwidth of 30 nm. Unlike ultraviolet (UV) light and other ionizing sources of radiation, the energy generated by the LEDs in this wavelength band is photothermal and does not have cumulative mutagenic potential. The clusters of LEDs were arranged over the inner surface of the helmet in order to provide 0.036 W/cm^2 of energy uniformly to the scalp. The

© 2020 Figueiro Longo MG et al. *JAMA Network Open.*

amount of light provided by the helmet was based on our prior animal experiments and is below the American National Standards Institute (ANSI) limit for maximum skin exposure of 0.1W/cm2 for 800nm NIR light.[1,2] Even though the wavelength composition of sun light is quite different, for comparison, the approximate solar irradiance at the Earth's surface is 0.1373 W/cm^2.[3]

A removable and disposable molded plastic piece isolated the patient form direct LED contact. Fans located on the exterior of the helmet dissipated any heat generated by the LEDs and the control electronics. The helmet was recalibrated after use on 10 subjects in order to ensure that the light intensity did not exceed the prespecified threshold of 36mW/cm^2 +/- 20%. The calibration was performed using a custom-built light monitoring device.

The LLLT helmet was used by a trained coordinator who also monitored and recorded the core temperature of each subject before and after each light therapy session. A control unit connected to the helmet by a 5-foot long cable was used to control the helmet state: On/Off and Active/Control. The control unit was kept hidden from the subject during the light therapy session.

MRI Acquisition and Processing

All MRI scans were performed on Siemens Prisma™ 3-Tesla scanners (Siemens Medical, Erlangen, Germany) using a 64-channel receiver head coil. The head was immobilized in the head coil with straps and foam pads. The whole brain MRI datasets for each scan consisted of standard high-resolution sagittal images acquired with volumetric T1-weighted 3D-MP-RAGE (TR=2530ms, TE=1.74ms / 3.6ms / 5.46ms / 7.32ms, flip angle=7º, FOV=256mm, matrix=256x256, slice thickness=1mm); a T2-SPACE-FLAIR sequence for the identification of possible brain lesions (TR=5000ms, TE=387ms, flip angle= T2 var, FOV=240mm, matrix=256x256, slice thickness=0.90mm); Susceptibility-weighted imaging (SWI) for detection of hemorrhages and microhemorrhages (TR=28ms, TE=20ms, flip angle=15, FOV=256mm, matrix=256x256, slice thickness=1.80mm); and diffusion tensor imaging (DTI; number of directions=96, b=0, 500, 1000, 2000 and 3000 s/mm^2, voxel size of 1.9mm×1.9mm×1.9mm). After the scanning, the DICOM images were stored in a Picture Archiving and Communications System (PACS) for off-line analysis.

Structural Data

We used the Freesurfer developmental version longitudinal stream to perform automated segmentation and cortical parcellation of the T1-weighted volumetric images.[4]

The longitudinal stream generates an unbiased, within-subject template for all three time-points of each subject using a robust, inverse consistent registration.[5] Several processing steps, such as skull stripping, Talairach transforms, atlas registration as well as spherical surface maps and parcellations were then

© 2020 Figueiro Longo MG et al. *JAMA Network Open.*

initialized with common information from the within-subject template, significantly increasing reliability and statistical power. All volumes were inspected for accuracy and minor manual edits were performed where needed by a trained operator. These were usually restricted to removal of non-brain tissue included within the cortical boundary.[4]

The longitudinal stream from the software TRActs Constrained by UnderLying Anatomy (TRACULA)[6] was used to automatically delineate following 18 major white matter tracts in each subject: corpus callosum – forceps major (F Major), corpus callosum – forceps minor (F Minor), anterior thalamic radiations (Left ATR and Right ATR), cingulum – angular bundles (Left CAB and Right CAB), cingulum – cingulate gyrus bundles (Left CCG and Right CCG), corticospinal tracts (Left CST and Right CST), inferior longitudinal fasciculi (Left ILF and Right ILF), superior longitudinal fasciculi – parietal terminations (Left SLFP and Right SLFP), superior longitudinal fasciculi – temporal terminations (Left SLFT and Right SLFT), and uncinate fasciculi (Left UNC and Right UNC).

TRACULA uses a global probabilistic algorithm that fits the shape of each tract both to the diffusion orientations at each voxel and to the prior information about the anatomical neighborhoods through which the tract should pass. TRACULA longitudinal stream uses the longitudinal diffusion data of each subjects as well as the anatomical neighborhoods from the subject's longitudinal T1-weighted images. The anatomical landmarks are segmented by the Freesurfer longitudinal stream.

Complete Study Protocol

The complete study protocol, as disclosed to the ClinicalTrials.gov, is provided in Appendix A.

Revised Power Calculation

Before the start of this clinical trial, the sample size was calculated using the data from a cohort of 12 moderate and severe TBI patients scanned using quantitative MRI (from Singh et al., 2010, Novel diffusion tensor imaging methodology to detect and quantify injured regions and affected brain pathways in traumatic brain injury. MRI 28 22-40). For this cohort, four DTI parameters --- namely, the fractional anisotropy (FA), the mean diffusivity (MD), the axial diffusivity L1 and the radial diffusivity (Lt) --- were measured in 16 regions of the brain. For this cohort of patients, the most variable DTI parameter was the FA: the coefficient of variation (CV), defined as the ratio of mean to the standard deviation, was 53.45%. The coefficients of variation of the other DTI parameters were smaller (MD: 18.34%, L1:16.44%, Lt: 22.43%).

Using the above values from the published literature, the required sample size for this study was

© 2020 Figueiro Longo MG et al. *JAMA Network Open.*

calculated using the following formula (obtained from: van Belle, G., 2002, Statistical Rules of Thumb, John Wiley & Sons Inc., p. 221):

$$n = 2 * [Z_{1-\alpha/2} + Z_{1-\beta}]^2 * (CV)^2 / (PC)^2$$

where: n = sample size in each group; PC = proportional change in mean values; and CV = coefficient of variation. The following values were used in the sample size calculation: alpha = 5% (i.e., at a significance level of 0.05); beta = 20% (i.e., with a statistical power of 0.8) and CV = 53.45% (estimated from the aforementioned study data). The required sample size for observing at least 25% difference (PC =25%) in DTI parameters between sham treatment vs. low-level light therapy, after 3 months of follow up, was estimated to be 144 (72 patients in each group).

Instead of using the parameters from published literature, we further recomputed the coefficient of variation CV using a cohort of scans available at our institution at the time of submission. The highest CV for this cohort was found to be for the fractional anisotropy (FA) parameter and had a value of 40%. Using CV=0.4 in the above calculation, we estimated the cohort size to be 82 patients, with 41 patients in the treatment arm and another 41 in the sham group.

Our current assessment of CV using the blinded data from the cohort we have enrolled so far reveals that the variability in our own cohort is in fact less than 40% that was used for estimating the cohort size at the time of grant submission. For the cerebral blood flow (CBF), measured using ASL, this value is about 25%. For the DTI parameters, the variability is even lower. Reduction in the parameter variability is most likely a result of using the same scanner by the same operator for all subjects. We also exercise extreme caution in care in positioning all patients in the same manner and making sure that all the parameters of the scan are tightly controlled.

If we substitute 0.25 for CV, instead of the original 0.40 obtained from other scans, the cohort size n reduces to 32. In order to be safe, and to account for data that may not be usable for various reasons (e.g., movement artifacts), we targeted a cohort of 40 patients before breaking the blind.

Supplemental Results

Effect of LLLT on Individual White Matter Tracts

In addition to the linear mixed effect model that tries to discern of effect of global variables (time, treatment, time x treatment), we also performed tract-by-tract analysis for each tract. The results are summarized in Figure S2.

© 2020 Figueiro Longo MG et al. *JAMA Network Open.*

In individuals in the light-treatment group, axial diffusivity (AD) was lower in 16/18 tracts at 2 weeks, and higher in 14/18 tracts at 3 months, compared to control group (binomial test, respectively, $p<0.01$ and $p = 0.03$). Similarly, at 2-weeks and 3-months, respectively, radial diffusivity (RD) was lower in 13/18 tracts and greater in 13/18 ($p=0.01$ for both), mean diffusivity (MD) was lower in 14/18 tracts and greater in 16/18 ($p=0.03$ and $p<0.01$), and functional anisotropy (FA) was lower in 14/18 tracts, and remained lower in 13/18 ($p=0.03$ and $p=0.10$) At the level of individual tracts, light had a small impact on diffusion parameters. The light induced effect did not reach statistical significance in most individual tracts even though the direction change was consistent across all tracts (Figure S2).

Random Effects Associated with the LME Model

The eTable 3 shows that variance components of the linear mixed effect model: population covariation (σ^2) between random factors and the dependent variable, and the variation (τ) and intraclass correlation coefficient (ICC) within random factors. N denotes the number of individual tracts and patients (ID).

© 2020 Figueiro Longo MG et al. *JAMA Network Open.*

eReferences

1. Smith M, Fork RL, Cole S. Safe delivery of optical power from space. Opt Express. 2001;8(10):537.

2. Sliney D, Wolbarsht M. Safety with Lasers and Other Optical Sources. (Press P, ed.). New York; 1980.

3. Lide DR. CRC Handbook of Chemistry and Physics. 79th editi. (CRC Press, ed.). Boca Raton, FL; 1998.

4. Reuter M, Schmansky N, Rosas HD, Fischl B. Within-subject template estimation for unbiased longitudinal image analysis. Neuroimage. 2012;61(4):1402-1418.

5. Reuter M, Rosas HD, Fischl B. Highly Accurate Inverse Consistent Robust Registration: A Robust Approach. Neuroimage. 2010;53(4):1181-1196.

6. Yendiki A, Reuter M, Wilkens P, Rosas H, Fischl B. Joint reconstruction of white-matter pathways from longitudinal diffusion MRI data with anatomical priors. Neuroimage. 2016;15(127):277-286.

© 2020 Figueiro Longo MG et al. *JAMA Network Open.*

eTable 1. Demographic and clinical characteristics of participants at enrollment into the study (N=68).

**Parameter**	**Total (N=68)**	**Sham Treatment (N=35)**	**Light Treatment (N=33)**	***p***
**Sex**				0.45
Female	30 (44.1%)	17 (48.6%)	13 (39.4%)	
Male	38 (55.9%)	18 (51.4%)	20 (60.6%)	
**Age**	49.71 (18.84)	52.76 (16.60)	46.47 (20.73)	0.18
**Injury Mechanism**				0.02
Bike/Motorcycle Accident With Helmet	4 (6.9%)	4 (13.8%)	0 (0.0%)	
Bike/Motorcycle Accident Without Helmet	1 (1.7%)	0 (0.0%)	1 (3.4%)	
Fall	32 (55.2%)	18 (62.1%)	14 (48.3%)	
Other	1 (1.7%)	1 (3.4%)	0 (0.0%)	
Pedestrian accident with car/motorcycle/bike	6 (10.3%)	3 (10.3%)	3 (10.3%)	
Restrained Automobile Accident	4 (6.9%)	0 (0.0%)	4 (13.8%)	
Unrestrained Automobile Accident	2 (3.4%)	2 (6.9%)	0 (0.0%)	
Violence/assault	8 (13.8%)	1 (3.4%)	7 (24.1%)	
**History**				
Hypertension	19 (27.9%)	10 (28.6%)	9 (27.3%)	0.91
Diabetes Mellitus (Type I or II)	7 (10.3%)	4 (11.4%)	3 (9.1%)	0.75
**Imaging Findings**				
Extracranial Hemorrhage	26 (38.2%)	11 (31.4%)	15 (45.5%)	0.23
Epidural Hemorrhage	2 (2.9%)	0 (0.0%)	2 (6.1%)	0.14
Acute Subdural Hemorrhage	21 (30.9%)	12 (34.3%)	9 (27.3%)	0.53
Subarachnoid Hemorrhage	25 (36.8%)	15 (42.9%)	10 (30.3%)	0.28
Edema	1 (1.5%)	1 (2.9%)	0 (0.0%)	0.33
Contusion	6 (8.8%)	2 (5.7%)	4 (12.1%)	0.35
Intraparenchymal Hemorrhage	10 (14.7%)	3 (8.6%)	7 (21.2%)	0.14

© 2020 Figueiro Longo MG et al. *JAMA Network Open.*

Intraventricular Hemorrhage	2 (2.9%)	0 (0.0%)	2 (6.1%)	0.14
Skull Fracture	15 (22.1%)	8 (22.9%)	7 (21.2%)	0.87
Intracranial Air	2 (2.9%)	1 (2.9%)	1 (3.0%)	0.97
Facial Fracture	16 (23.5%)	10 (28.6%)	6 (18.2%)	0.31
Orbital Injury	6 (8.8%)	3 (8.6%)	3 (9.1%)	0.94
**Fazekas scale:** Periventricular White Matter Hyperdensities				0.44
Absent (0) / "Caps" or pencil-thin lining (1)	37 (88.1%)	19 (82.6%)	18 (94.7%)	
Smooth "halo" (2)	4 (9.5%)	3 (13.0%)	1 (5.3%)	
Irregular periventricular signal extending into the deep white matter (3)	1 (2.4%)	1 (4.3%)	0 (0.0%)	
**Fazekas scale:** Deep White Matter Hyperdensities				0.64
Absent (0) / Punctate foci (1)	35 (83.3%)	19 (82.6%)	16 (84.2%)	
Beginning confluence (2)	6 (14.3%)	3 (13.0%)	3 (15.8%)	
Large confluent areas (3)	1 (2.4%)	1 (4.3%)	0 (0.0%)	
**Hypertension**	19 (27.9%)	10 (28.6%)	9 (27.3%)	0.91
**Diabetes Mellitus**	7 (10.3%)	4 (11.4%)	3 (9.1%)	0.75
**Hospital Course**				
AED*	38 (55.9%)	21 (60.0%)	17 (51.5%)	0.48
Physical Therapy	29 (42.6%)	16 (45.7%)	13 (39.4%)	0.60
Occupational Therapy	34 (50.0%)	24 (68.6%)	10 (30.3%)	0.92
Speech Therapy	5 (7.4%)	4 (11.4%)	1 (3.0%)	0.19
Rehabilitation	4 (5.9%)	4 (11.4%)	0 (0.0%)	0.05
ICU Stay	6 (8.8%)	4 (11.4%)	2 (6.1%)	0.44
**Clinical Signs and Symptoms**				
RPQ-3	4.38 (3.44)	5.50 (3.45)	3.12 (3.03)	0.01
RPQ-13	11.35 (8.78)	12.11 (7.99)	10.46 (9.72)	0.50
RPQ Total	15.73 (10.78)	17.61 (9.78)	13.54 (11.67)	0.18

*AED : anti-epileptic drug prophylaxis

Continuous variables (RPQ scores and age) are presented as mean (standard deviation).

© 2020 Figueiro Longo MG et al. *JAMA Network Open.*

eTable 2. Summary of the brain diffusion parameters based on the time-point and treatment group

Timepoint	Parameter	Group	N	Mean	SD	SE	CI
Baseline	AD	Control	413	0.8259	0.1712	0.0084	0.0166
Baseline	AD	Light Treatment	252	0.8536	0.1803	0.0114	0.0224
Baseline	FA	Control	413	0.4739	0.0984	0.0048	0.0095
Baseline	FA	Light Treatment	252	0.4820	0.1045	0.0066	0.0130
Baseline	MD	Control	413	0.5259	0.1192	0.0059	0.0115
Baseline	MD	Light Treatment	252	0.5391	0.1168	0.0074	0.0145
Baseline	RD	Control	413	0.3760	0.1087	0.0053	0.0105
Baseline	RD	Light Treatment	252	0.3819	0.1056	0.0067	0.0131
2-week	AD	Control	378	0.8115	0.1496	0.0077	0.0151
2-week	AD	Light Treatment	304	0.8331	0.1653	0.0095	0.0187
2-week	FA	Control	378	0.4755	0.0992	0.0051	0.0100
2-week	FA	Light Treatment	304	0.4727	0.1033	0.0059	0.0117
2-week	MD	Control	378	0.5154	0.1017	0.0052	0.0103
2-week	MD	Light Treatment	304	0.5298	0.1047	0.0060	0.0118
2-week	RD	Control	378	0.3673	0.0960	0.0049	0.0097
2-week	RD	Light Treatment	304	0.3781	0.0962	0.0055	0.0109
3-month	AD	Control	396	0.8270	0.1686	0.0085	0.0167
3-month	AD	Light Treatment	287	0.8367	0.1649	0.0097	0.0192
3-month	FA	Control	396	0.4847	0.1166	0.0059	0.0115
3-month	FA	Light Treatment	287	0.4665	0.1019	0.0060	0.0118
3-month	MD	Control	396	0.5201	0.1098	0.0055	0.0109
3-month	MD	Light Treatment	287	0.5351	0.1053	0.0062	0.0122
3-month	RD	Control	396	0.3667	0.1060	0.0053	0.0105
3-month	RD	Light Treatment	287	0.3844	0.0968	0.0057	0.0112

© 2020 Figueiro Longo MG et al. *JAMA Network Open.*

eTable 3. Random effects and other statistical parameters derived from the linear mixed effect model. ICC for nested models shows the proportion of variance explained for each grouping level.

**Random Effects**	**AD**	**RD**	**MD**	**FA**
σ^2	0.00	0.00	0.00	0.00
τ_{00}	0.00 $_{tract:ID}$	0.01 $_{tract:ID}$	0.00 $_{tract:ID}$	0.01 $_{tract:ID}$
	0.02 $_{ID}$	0.00 $_{ID}$	0.01 $_{ID}$	0.00 $_{ID}$
ICC	0.89	0.93	0.92	0.89
N	18 $_{tract}$	18 $_{tract}$	18 $_{tract}$	18 $_{tract}$
	42 $_{ID}$	42 $_{ID}$	42 $_{ID}$	42 $_{ID}$
Observations	2030	2030	2030	2030

© 2020 Figueiro Longo MG et al. *JAMA Network Open.*

eFigure 1-LLLT helmet

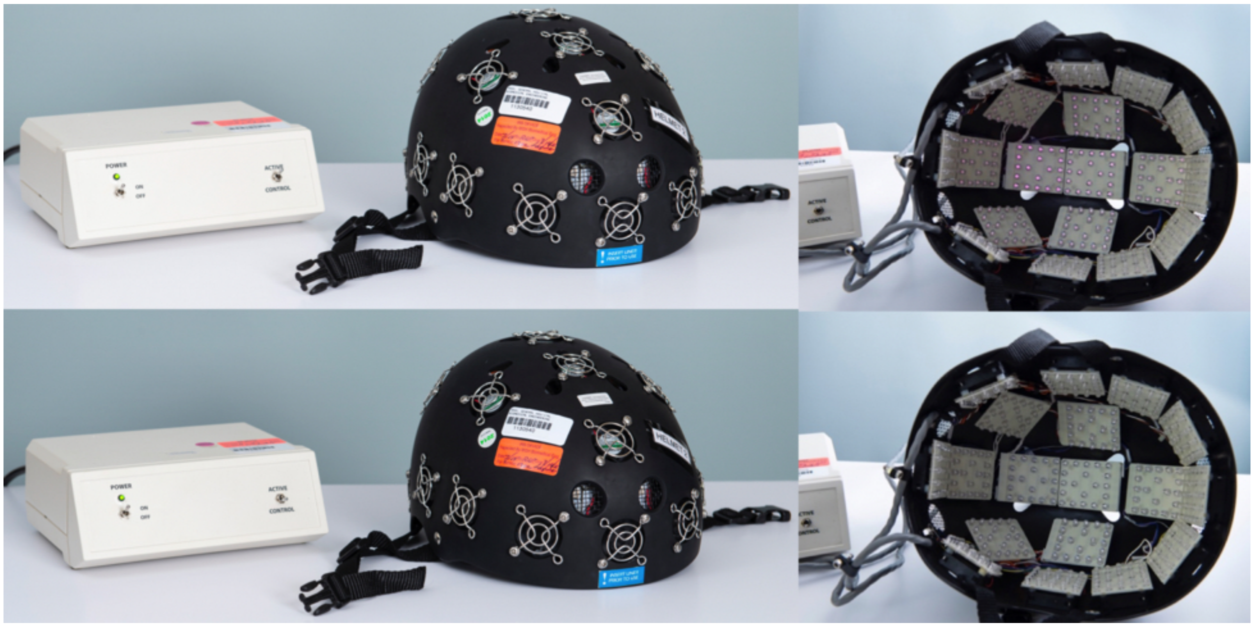

© 2020 Figueiro Longo MG et al. *JAMA Network Open.*

eFigure 2-Temporal evolution of each of the 18 individual white matter tracts

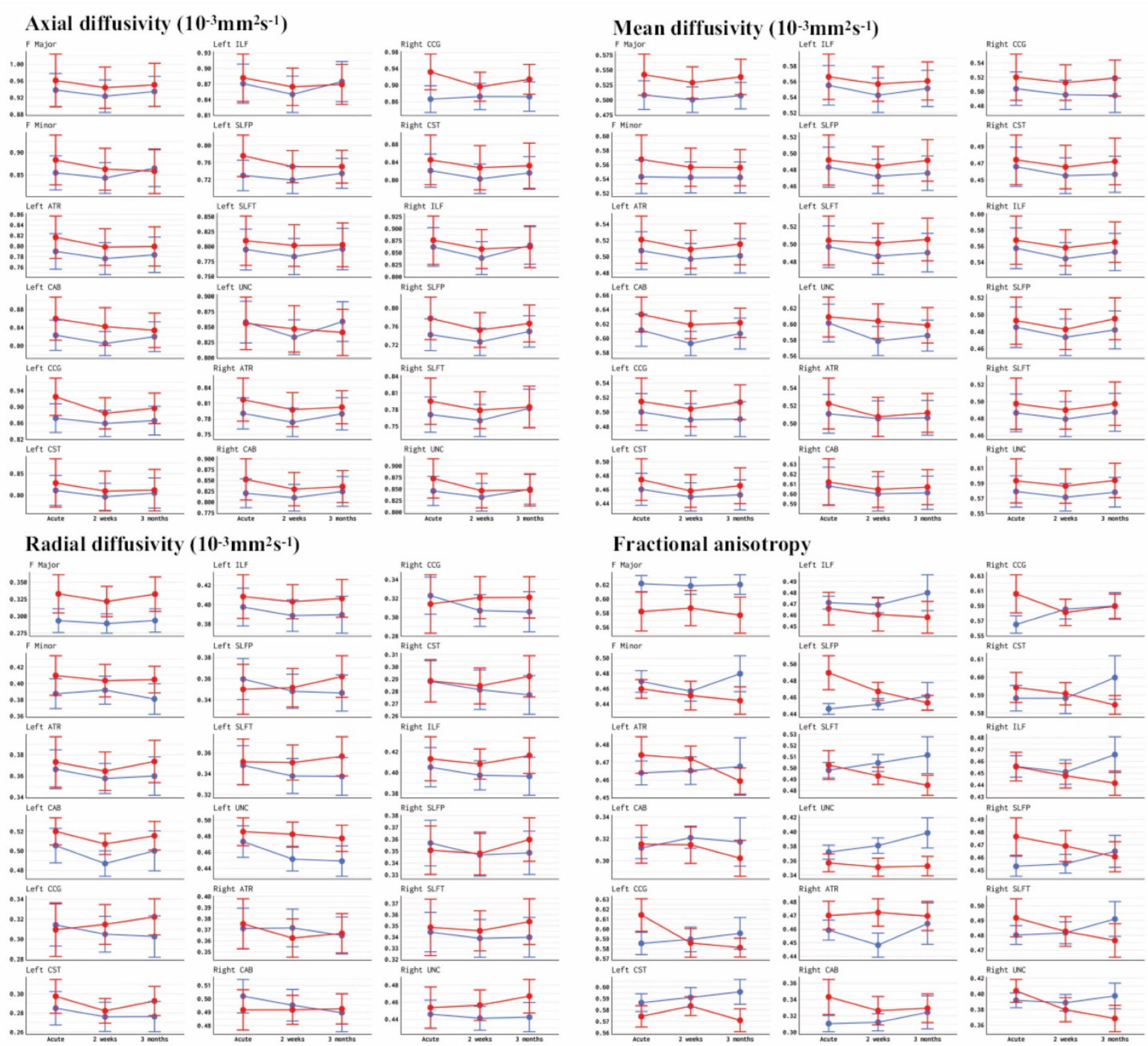

© 2020 Figueiro Longo MG et al. *JAMA Network Open.*
