# Supplementary material for: Effect of Transcranial Low-Level Light Therapy vs Sham Therapy Among Patients With Moderate Traumatic Brain Injury: A Randomized Clinical Trial
Source: JAMA Netw Open. 2020 Sep 14;3(9):e2017337. doi: 10.1001/jamanetworkopen.2020.17337 (PMC7490644; doi:10.1001/jamanetworkopen.2020.17337)
Supplement: Supplement 3. — Data Sharing Statement [file jamanetwopen-e2017337-s003.pdf]

Data Sharing Statement

Data

Data available: Yes
Data types: Deidentified participant data
How to access data: mfigueirolongo@mgh.harvard.edu
When available: With publication

Supporting Documents

Document types: Statistical/analytic code, Informed consent form
How to access documents: mfigueirolongo@mgh.harvard.edu
When available: With publication

Additional Information

Who can access the data: researchers whose proposed use of the data has been approved
Types of analyses: comparison betwee results
Mechanisms of data availability: researchers whose proposed use of the data has been approved
